# Inhibition of the NLRP3/IL‐1β axis protects against sepsis‐induced cardiomyopathy

**DOI:** 10.1002/jcsm.12763

**Published:** 2021-09-02

**Authors:** Katharina Busch, Melanie Kny, Nora Huang, Tilman E. Klassert, Magdalena Stock, Alexander Hahn, Sebastian Graeger, Mihail Todiras, Sibylle Schmidt, Bishwas Chamling, Michael Willenbrock, Stefan Groß, Doreen Biedenweg, Arnd Heuser, Claus Scheidereit, Christian Butter, Stephan B. Felix, Oliver Otto, Friedrich C. Luft, Hortense Slevogt, Jens Fielitz

**Affiliations:** ^1^ Experimental and Clinical Research Center (ECRC), Charité‐Universitätsmedizin Berlin, Max Delbrück Center (MDC) for Molecular Medicine in the Helmholtz Association Berlin Germany; ^2^ Department of Cardiology Heart Center Brandenburg and Medical School Brandenburg (MHB) Bernau Germany; ^3^ ZIK Septomics, Host Septomics Jena Germany; ^4^ Jena University Hospital, Integrated Research and Treatment Center ‐ Center for Sepsis Control and Care (CSCC) Jena Germany; ^5^ DZHK (German Center for Cardiovascular Research), Partner Site Greifswald Greifswald Germany; ^6^ Laboratory of Molecular Biology of Peptide Hormones Max Delbrück Center for Molecular Medicine Berlin Germany; ^7^ Nicolae Testemiţanu State University of Medicine and Pharmacy Chișinău Moldova; ^8^ Department of Internal Medicine B, Molecular Cardiology University Medicine Greifswald Greifswald Germany; ^9^ Signal Transduction in Development and Cancer Max Delbrück Center for Molecular Medicine Berlin Germany; ^10^ Centre for Innovation Competence ‐ Humoral Immune Response in Cardiovascular Diseases University of Greifswald Greifswald Germany; ^11^ Animal Phenotyping Facility Max Delbrück Center for Molecular Medicine Berlin Germany

**Keywords:** NLR family, pyrin domain‐containing 3 protein, Interleukin‐1 beta, Sepsis, Heart failure

## Abstract

**Background:**

Septic cardiomyopathy worsens the prognosis of critically ill patients. Clinical data suggest that interleukin‐1β (IL‐1β), activated by the NLRP3 inflammasome, compromises cardiac function. Whether or not deleting *Nlrp3* would prevent cardiac atrophy and improve diastolic cardiac function in sepsis was unclear. Here, we investigated the role of NLRP3/IL‐1β in sepsis‐induced cardiomyopathy and cardiac atrophy.

**Methods:**

**Male** *Nlrp3* knockout (KO) and wild‐type (WT) mice were exposed to polymicrobial sepsis by caecal ligation and puncture (CLP) surgery (KO, *n* = 27; WT, *n* = 33) to induce septic cardiomyopathy. Sham‐treated mice served as controls (KO, *n* = 11; WT, *n* = 16). Heart weights and morphology, echocardiography and analyses of gene and protein expression were used to evaluate septic cardiomyopathy and cardiac atrophy. IL‐1β effects on primary and immortalized cardiomyocytes were investigated by morphological and molecular analyses. IonOptix and real‐time deformability cytometry (RT‐DC) analysis were used to investigate functional and mechanical effects of IL‐1β on cardiomyocytes.

**Results:**

Heart morphology and echocardiography revealed preserved systolic (stroke volume: WT sham vs. WT CLP: 33.1 ± 7.2 μL vs. 24.6 ± 8.7 μL, *P* < 0.05; KO sham vs. KO CLP: 28.3 ± 8.1 μL vs. 29.9 ± 9.9 μL, n.s.; *P* < 0.05 vs. WT CLP) and diastolic (peak E wave velocity: WT sham vs. WT CLP: 750 ± 132 vs. 522 ± 200 mm/s, *P* < 0.001; KO sham vs. KO CLP: 709 ± 152 vs. 639 ± 165 mm/s, n.s.; *P* < 0.05 vs. WT CLP) cardiac function and attenuated cardiac (heart weight–tibia length ratio: WT CLP vs. WT sham: −26.6%, *P* < 0.05; KO CLP vs. KO sham: −3.3%, n.s.; *P* < 0.05 vs. WT CLP) and cardiomyocyte atrophy in KO mice during sepsis. IonOptix measurements showed that IL‐1β decreased contractility (cell shortening: IL‐1β: −15.4 ± 2.3%, *P* < 0.001 vs. vehicle, IL‐1RA: −6.1 ± 3.3%, *P* < 0.05 vs. IL‐1β) and relaxation of adult rat ventricular cardiomyocytes (time‐to‐50% relengthening: IL‐1β: 2071 ± 225 ms, *P* < 0.001 vs. vehicle, IL‐1RA: 564 ± 247 ms, *P* < 0.001 vs. IL‐1β), which was attenuated by an IL‐1 receptor antagonist (IL‐1RA). RT‐DC analysis indicated that IL‐1β reduced cardiomyocyte size (*P* < 0.001) and deformation (*P* < 0.05). RNA sequencing showed that genes involved in NF‐κB signalling, autophagy and lysosomal protein degradation were enriched in hearts of septic WT but not in septic KO mice. Western blotting and qPCR disclosed that IL‐1β activated NF‐κB and its target genes, caused atrophy and decreased myosin protein in myocytes, which was accompanied by an increased autophagy gene expression. These effects were attenuated by IL‐1RA.

**Conclusions:**

IL‐1β causes atrophy, impairs contractility and relaxation and decreases deformation of cardiomyocytes. Because NLRP3/IL‐1β pathway inhibition attenuates cardiac atrophy and cardiomyopathy in sepsis, it could be useful to prevent septic cardiomyopathy.

## Introduction

Sepsis is the leading cause of death of critically ill patients, and septic cardiomyopathy contributes to this outcome.^1^ Cardiomyopathy is found in half of septic patients.^2^ The problem is underappreciated, possibly due to the observation that the left ventricular ejection fraction (LVEF) in septic patients is not invariably reduced from normal values and does not reach the degree found in heart failure (HF). To assure an adequate perfusion pressure during sepsis, a strong increase in cardiac output is required. If this increase is insufficient to meet metabolic needs, the diagnosis of septic cardiomyopathy is established. The mortality of septic patients is directly associated with systolic^3^ and diastolic^4^ cardiac dysfunction, indicating that the prognosis of critically ill septic patients rests on global cardiac dysfunction.^5^ Because cardiac function normalizes within 2 weeks after sepsis, functional rather than structural cardiac abnormalities have been implicated.^6^

Cardiodepressant inflammatory cytokines, particularly interleukin‐1β (IL‐1β) and tumour necrosis factor (TNF), are significantly increased during sepsis in both patients and animal models.^7^ Importantly, an IL‐1β antagonist attenuated the haemodynamic and metabolic manifestations of septic shock.^8^ IL‐1β was reported to decrease myocardial contractility *in vitro* and *ex vivo*.^9^, ^10^ IL‐1β and TNF reduced cardiomyocyte function *in vitro*, when cardiomyocytes were incubated with serum from septic shock patients, and IL‐1β immunoabsorption diminished the cardiodepressant activity of this serum.^11^ Nevertheless, structural abnormalities in cardiomyocytes affecting sarcomeres, mitochondria and sarcoplasmic reticula appear to be similarly important for septic cardiomyopathy.^12^ IL‐1β expression, maturation and secretion are tightly controlled. Pro‐IL‐1β is the inactive IL‐1β precursor; pro‐IL‐1β conversion to IL‐1β and IL‐1β‐secretion are mediated by inflammasomes. These multiprotein complexes act as scaffolds for caspase‐1‐mediated maturation and secretion of IL‐1β and IL‐18.^13^ Inflammasomes are involved in sepsis and septic muscle failure.^14^ Cytoplasmic receptors of the nucleotide‐binding domain (NOD)‐like receptor (NLR) family are key inflammasome components.^13^ The NOD‐, leucine‐rich repeat (LRR)‐ and pyrin domain‐containing protein 3 (NLRP3) is one of the best characterized NLR proteins capable of forming an inflammasome complex. NLRP3 is contained in the heart^14^, ^15^, ^16^ and skeletal muscle. Inflammation in general and the NLRP3 inflammasome in particular are involved in the pathophysiology of cardiovascular diseases,^17^ including HF^18^ and myocardial ischemia.^19^ All components of the NLRP3 inflammasome are expressed in the heart albeit at lower levels compared to tissues that are involved in immune defence.^20^ However, myocardial NLRP3 and IL‐1β were shown to be upregulated in response to myocardial ischemia.^19^ Recent studies have reported that inhibition of the inflammasome may be useful to prevent ischemia‐induced myocardial damage.^19^ More specifically, inhibition of the NLRP3 inflammasome significantly reduced the infarct size and preserved cardiac function that accompany myocardial ischemia.^21^ It has also been shown that Ca2+/Calmodulin‐dependent protein kinase II (CaMKII) δ‐mediated activation of the NLRP3 inflammasome in cardiomyocytes mediates fibrosis and cardiac dysfunction in response to chronic pressure overload.^22^ The NLRP3 inflammasome/CaMKII pathway is also implicated in post‐operative atrial fibrillation in atrial myocytes from patients.^23^ These data indicate that the NLRP3 inflammasome is involved in inflammation‐induced myocardial pathologies.

Consequently, several groups used *Nlrp3* knockout (KO) mice and/or NLRP3 inhibitors to investigate if the NLRP3 inflammasome is also involved in sepsis‐induced HF.^15^, ^24^, ^25^, ^26^, ^27^, ^28^, ^29^, ^30^ Interestingly, the NLRP3 inflammasome inhibitors melatonin and cortistatin showed favourable effects in sepsis‐induced cardiac dysfunction in mice.^26^, ^27^ Although an activation of the NLRP3 inflammasome was reported to mediate cardiac electrical and functional defects in low‐ and medium‐grade sepsis induced by the caecal ligation and puncture (CLP),^25^ this could not be found in high‐grade polymicrobial sepsis induced by the colon ascendens stent peritonitis (CASP) model.^24^ Additionally, it is uncertain if deletion of the NLRP3 inflammasome improves both systolic and diastolic dysfunction in sepsis. Although echocardiography was performed by some groups 8,^15^, ^25^ 18^30^ or 24 h^24^ after CLP surgery, parameters of diastolic function were not reported by all. In addition, not all groups performed echocardiography to evaluate cardiac function in sepsis.^25^, ^27^, ^28^, ^29^ Importantly in their work Kalbitz et al.^15^ showed a trend towards a better diastolic function in *Nlrp3* KO mice compared with controls, which did not reach statistical significance 8 h after CLP surgery.^15^ Finally, whether or not deletion of *Nlrp3* attenuates cardiac atrophy in sepsis has not been investigated.

Because we have previously shown that IL‐1β mediates skeletal muscle atrophy *in vivo* and myocyte atrophy *in vitro*,^31^ we hypothesized that this pro‐inflammatory cytokine also targets cardiomyocytes and the heart during sepsis. We investigated if deletion of *Nlrp3* inhibits sepsis‐induced cardiac atrophy and if this is accompanied by an improved diastolic and systolic cardiac function in medium‐grade sepsis after 24 h. To test if IL‐1β is mechanistically involved in septic cardiomyopathy, we performed a novel high‐throughput method called real‐time deformability assay to determine if IL‐1β causes atrophy and affects the mechanical properties of cardiomyocytes. We further analysed if IL‐1β via its receptor causes atrophy and decreases relaxation and contractility of cardiomyocytes. Finally, we performed next‐generation sequencing of RNAs isolated from the interventricular septum to identify pathways involved in septic cardiomyopathy.

## Methods

### Animal model of polymicrobial sepsis

All animal procedures were performed in accordance with the guidelines of the Max Delbrück Center for Molecular Medicine and the Charité‐Universitätsmedizin Berlin and were approved by the Landesamt für Gesundheit und Soziales (LAGeSo, Berlin, Germany) for the use of laboratory animals (permit number G 207/13). The investigation conforms to the *Guide for the Care and Use of Laboratory Animals* published by the US National Institutes of Health (NIH Publication No. 85‐23, revised 1985), as well as the current version of German law on the protection of animals. *Nlrp3* KO mice were kindly provided by Aubry Tardivel and Nicolas Fasel of the University of Lausanne.^32^ Genotyping was performed as recently described^31^ using primer pairs shown in *Table* S1. CLP surgery was performed to induce polymicrobial sepsis in 12‐ to 16‐week‐old male *Nlrp3* KO or *Nlrp3* wild‐type (WT) mice as recently described.^31^, ^33^ Sham mice were treated identically except for the ligation and puncture of the caecum. Mice were sacrificed 96 h after surgery.

### Two‐dimensional transthoracic echocardiography

Two‐dimensional transthoracic echocardiography was performed by two blinded technicians before and 24 h after CLP or sham surgery and analysed by one blinded physician (AH) as previously described.^34^ Briefly, mice were anaesthetized with 2% isoflurane and kept warm on a heated platform. Temperature and electrocardiography were continuously monitored. Systolic and diastolic cardiac function and morphology were assessed with a VisualSonics Vevo 2100 High‐Resolution Imaging System with the use of a high‐resolution (38‐MHz) transducer. Left ventricular (LV) end‐diastolic (LVIDd [mm]) and end‐systolic dimension (LVIDs [mm]), thickness of the left ventricular posterior wall in diastole (LVPWthd [mm]) and systole (LVPWths [mm]), thickness of the interventricular septum in diastole (IVSthd [mm]) and systole (IVSths [mm]) and fractional shortening (FS, %) were measured, and LVEF (%), cardiac output (CO [ml/min]) and stroke volume (SV [μl]) were calculated. Peak mitral inflow E (mm/s) and A (mm/s) velocity waves on pulsed‐wave Doppler were measured from the apical four‐chamber view. The ratio of peak flow velocity across the mitral annulus during early and late diastole (E/A) was calculated from these values as metric of diastolic dysfunction.

### Isolation of adult ventricular rat cardiomyocytes and measurement of cell shortening/relengthening

Isolation of ventricular cardiomyocytes from the hearts of adult Wistar rats (adult rat ventricular cardiomyocytes [ARVM]) was performed by retrograde collagenase perfusion of the excised heart in a Langendorff apparatus as recently described.^35^ Following incremental calcium restoration, freshly isolated ARVM were suspended in buffer A (117 mM NaCl, 2.8 mM KCl, 0.6 mM MgCl_2_, 1.2 mM KH_2_PO_4_, 1.2 mM CaCl_2_, 20 mM glucose, 10 mM HEPES (2‐[4‐(2‐hydroxyethyl)‐1‐piperazinyl]‐ethanesulfonic acid), pH 7.3), plated onto laminin‐coated wells (laminin from Engelbreth–Holm–Swarm murine sarcoma basement membrane, Sigma‐Aldrich) and allowed to adhere for 1 h at room temperature. Only rod‐shaped cells that showed clear striations and no spontaneous contractions were used for further analysis. Mechanical properties of cardiomyocytes were assessed using a SoftEdgeMyoCam® system (IonOptix Corporation, Milton, MA, USA) as described previously.^35^ After measurements of basal contraction, ARVM were treated with vehicle (PBS), IL‐1β (10 ng/mL, Sigma‐Aldrich) and an IL‐1 receptor antagonist (IL‐1RA) (10 μg/mL, Sigma‐Aldrich; 30 min prior to IL‐1β treatment). Intracellular Ca^2+^ was measured and recorded using a dual‐excitation, single‐emission photomultiplier system (IonOptix) in ARVM loaded with the calcium fluorescent probe Fura‐2‐AM (Sigma‐Aldrich) and during field stimulation of single ARVM (1 Hz, 5 ms). Changes in intracellular calcium transients were inferred from the ratio of fluorescence intensities excited at 340 and 380 nm. For calculation of changes in systolic cell shortening and calcium transients, cell length and Fura‐2‐AM fluorescence of the monitored cell were measured after a 2‐min equilibrium period and again after 5 min. All data are shown as percentage change from baseline. At least 10 different ARVM from at least six different isolations were measured and used for calculations. The IonOptix SoftEdge software was used to capture changes in cell length during shortening and relengthening. Shortening and relengthening of ARVM were assessed using the following indices: cell shortening (the amplitude myocytes shortened upon electrical stimulation), indicative for peak ARVM contractility; time‐to‐50% relengthening, the duration to reach 50% relengthening, an indicative of diastolic duration (50% rather 100% relengthening was used to avoid noisy signal at baseline level); and change of relaxation speed, indicative for the velocity of ARVM relaxation. The negative inotropic activity (NIA) of IL‐1β was defined as its ability to decrease cell shortening more than the mean effect induced by vehicle minus twice the standard deviation (mean − 2× SD).^35^ Because, vehicle (PBS) reduced the contractility of ARVM by 3.98 ± 0.80%, we defined the threshold for NIA as −9.05% from baseline.

### Real‐time deformability cytometry

In the last decades, mechanical properties of cells have been proven highly relevant to characterize cellular function assumption‐free without the need of antibodies or fluorescent markers. Here, mechanical properties of cells were measured by real‐time deformability cytometry (RT‐DC) as described elsewhere^36^ using the AcCelerator (Zellmechanik Dresden, Germany), a device that utilizes a 30 × 30 μm cross‐sectional constriction within a microfluidic chip to measure the hydrodynamic deformation of cells. Cell size and deformation were quantified using high‐speed image analysis of up to 1000 cells per second in real time. Prior to the experiment, HL‐1 cells were treated with recombinant IL‐1β (50 ng/mL) for 24 h. The IL‐1RA was added 30 min prior to IL‐1β treatment. After treatment, cells were washed with PBS and incubated with Trypsin‐EDTA (Sigma‐Aldrich) for 1 min at room temperature. Detached cells were centrifuged at 500 × g for 5 min and resuspended in 0.6% (*w*/*v*) methylcellulose in Mg^2+^‐ and Ca^2+^‐free PBS. The prefilled microfluidic chip was flushed with the cell suspension, and the flow was stabilized at 0.16 μL/s for 1 min before measurements. For each condition, more than 1000 mechanical single‐cell measurements were acquired. Material properties have been extracted applying an analytical model that utilized the full flow profile around a cardiomyocyte moving in the constriction of the microfluidic system. Coupling the hydrodynamic stress profile around the cell to linear elasticity theory and solving the inverse problem enables decoupling of cell size and deformation and calculation of a Young modulus. Data analysis was carried out based on experimental triplicates from three different days using linear mixed models.^37^ Briefly, by performing RT‐DC on biological replicates each consisting of several thousands of cells, the application of linear mixed models allows for separation of random and fixed effects in these large datasets. Fixed effects represent the quantity of interest, whereas random effects summarize all systematic and random measurement bias. Practically, two models are stated, one with and one without the fixed‐effect term, and statistical significance is calculated using Wilks' theorem.

### RNA sequencing and statistical analyses

For RNA sequencing, total RNA was isolated from the interventricular septum of three biological replicates (*n* = 3 mice per group; i.e. *Nlrp3* WT sham, *Nlrp3* WT CLP, *Nlrp3* KO sham, *Nlrp3* KO CLP) using TRIzol® Reagent (Invitrogen™, Life Technologies Corporation, CA, USA) and the FastPrep‐24™ instrument (MP Biomedicals GmbH) in accordance with manufacturer's instructions. RNAs were sequenced using the Ion Torrent proton™ sequencing technology. In total, three sequencing runs were accomplished, each multiplexed with one replicate of each sample type. The raw sequence data in FASTQ‐format are stored in the Sequence Read Archive (SRA) at the *National Center for Biotechnology Information* (NCBI) under the accession number SRP00123 (https://www.ncbi.nlm.nih.gov/). The genes of interest that were differentially expressed following CLP operation (using the contrast option of DESeq2 for CLP vs. sham) were chosen for further analysis. Using the DAVID tool, the upregulated genes as well as the downregulated genes were annotated using the KEGG (Kyoto Encyclopedia of Genes and Genomes) database to identify enriched categories, respectively. For the most relevant enriched categories, heatmaps of their assigned genes were generated. Those heatmaps provide useful information about those genes of these categories that were differently expressed in *Nlrp3* WT and *Nlrp3* KO mice.

### Statistical tests

All experiments were performed independently and at least three times using biological triplicates each. One‐way ANOVA with post hoc correction (Tukey's post‐comparison test) was used to analyse all qPCR data from animal and cell culture samples. Paired *t*‐test was used to study the distribution of myotube diameter in H9c2 myotubes. Differences were considered statistically significant at *P* ≤ 0.05. Data are shown as mean ± standard error of the mean (SEM) or standard deviation (SD) as indicated in bar plots. The GraphPad Prism® 6 program (GraphPad Software, La Jolla, CA, USA), Adobe Illustrator CS6, Version 16.0.0, and Photoshop CS6, Version 13.0, were used to perform statistics and draw graphics, respectively. The data obtained from measurements of cell shortening are expressed as percentwise change from baseline, and all values are given as mean ± SEM. Calculations were performed with Stata 14.1 (StataCorp. LP, College Station, USA), with a significance level of *P* ≤ 0.05. For statistical analyses of RNA sequencing data, please refer to *Methods*. Figures were drawn by Prism (Version 5). The documentation of immunofluorescence and histological staining results were performed with a Leica fluorescence microscope using Leica cameras (DFC360 FX and DFC425) and the LAS.AF software (version: 2.4.1 build 6384) and the LAS3.1 software (Version 2.5.0.6735).

For routine techniques, such as histological and immunohistochemical analyses, measurements of serum IL‐1β, myoblast culture, differentiation and atrophy assay, RNA isolation, cDNA synthesis and quantitative real‐time‐PCR, protein extraction, Western blot assay and EMSA and more information about RNA sequencing and statistical analyses, please refer to Supporting Information.

## Results

### *Nlrp3* deletion protects against septic cardiomyopathy and reduces NF‐κB signalling

**Figure 1:**
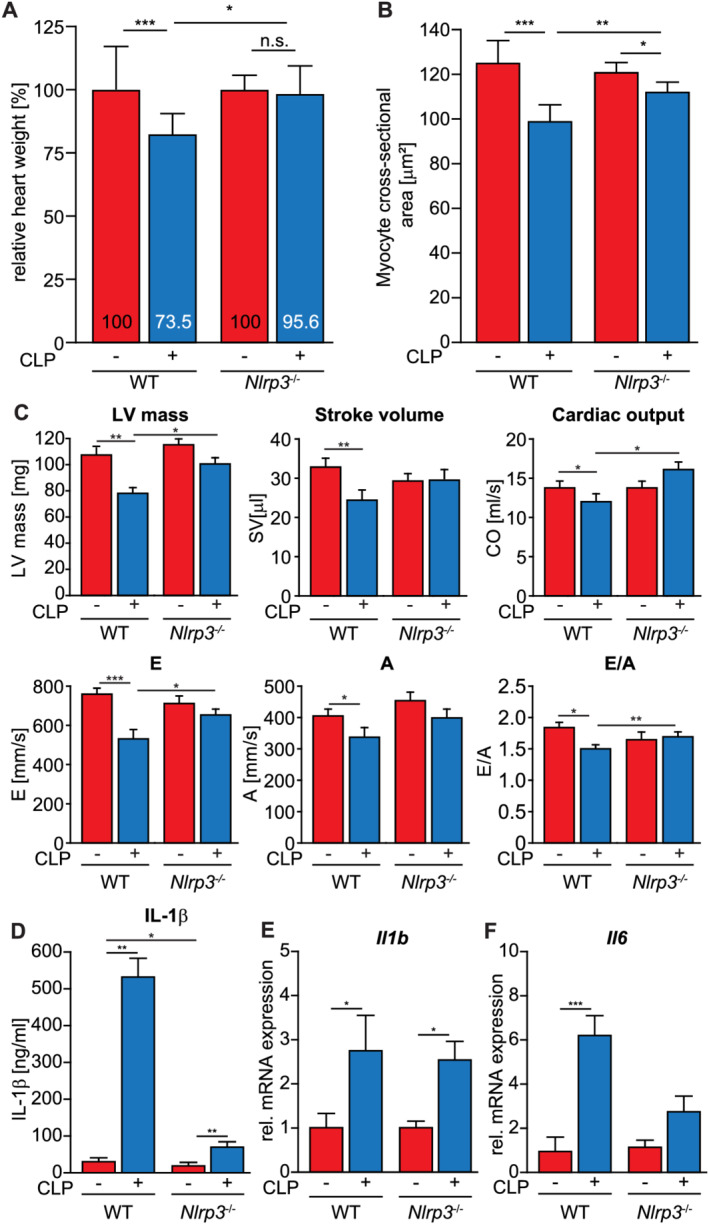
*Nlrp3* KO mice show less cardiac atrophy and a preserved cardiac function during sepsis. 12‐ to 16‐week‐old male *Nlrp3* KO and WT mice were subjected to CLP or sham surgery. *(A)* Heart weight normalized to tibia length and expressed as percent‐wise change compared with the respective sham group 96 h after CLP or sham surgery. CLP‐treated *Nlrp3* KO (*n* = 16); sham *Nlrp3* KO (*n* = 8), CLP *Nlrp3* WT (*n* = 12), sham *Nlrp3* WT (*n* = 13). *(B)* Mean myocyte cross‐sectional area (MCSA) determined in haematoxylin and eosin‐stained histological cross sections from hearts of CLP‐treated *Nlrp3* KO (*n* = 6), sham *Nlrp3* KO (*n* = 6), CLP *Nlrp3* WT (*n* = 6) and sham *Nlrp3* WT (*n* = 6) mice (*n* = 100 myocytes per mouse). Data are presented as mean ± SEM. *P ≤ 0.05; **P ≤ 0.01; ^***^ *P* ≤ 0.001. *(C)* Results of transthoracic echocardiography of Nlrp3 WT sham (n = 13), Nlrp3 WT CLP (n = 13), Nlrp3 KO sham (n = 18) and Nlrp3 KO CLP (n = 18) mice 24 h after CLP or sham operation. *(D)* Serum IL‐1β concentration in sham and CLP mice. Nlrp3 WT sham (n = 15), CLP Nlrp3 WT (n = 12), Nlrp3 KO sham (n = 14), Nlrp3 KO CLP (n = 14). Data are presented as mean ± SEM. *P ≤ 0.05; **P ≤ 0.01. qRT‐PCR analysis of *Il1b* *(E)* and *Il6* *(F)* expression in hearts of *Nlrp3* WT sham (*n* = 13), *Nlrp3* WT CLP (*n* = 12), *Nlrp3* KO sham (*n* = 8) and *Nlrp3* KO CLP (*n* = 15) mice. mRNA expression was normalized to *Gapdh*. Data are presented as mean ± SEM. *P ≤ 0.05; **P ≤ 0.01; ^***^ *P* ≤ 0.001; n.s., not significant.

We subjected *Nlrp3* KO and *Nlrp3* WT mice to CLP (KO, *n* = 27; WT, *n* = 33) and sham surgery (KO, *n* = 11; WT, *n* = 16). Compared with WT mice, significantly less KO mice died after CLP surgery after 96 h (57.6 vs. 29.6%; *P* < 0.05). Septic WT but not KO mice showed a significant reduction in body, lung and liver weights (*Table* S2). Heart weight‐to‐tibia length (HW/TL) ratio (*Figure* 1A) was significantly decreased in septic WT compared with sham mice (7.01 ± 1.23 vs. 5.15 ± 1.00 mg/mm; *P* < 0.02). In contrast, the HW/TL ratio was not different between sham‐ and CLP‐treated KO mice (6.71 ± 0.65 vs. 6.49 ± 1.09 mg/mm; *P* = 0.53), indicating that KO mice were protected from cardiac atrophy. Analyses of haematoxylin–eosin and trichrome staining of histological cross sections showed no interstitial fibrosis, irrespective of genotype and treatment (*Figure* S1A–D). However, measurements of myocyte cross‐sectional area (MCSA) revealed that myocytes of septic WT mice were significantly smaller when compared with sham‐treated WT mice. Although a reduction in MCSA was also observed in septic KO compared with sham‐treated KO mice, this reduction was significantly smaller when compared with MCSAs of septic WT mice (*Figure* 1B).

Echocardiography (*Figure* 1C) showed a decreased LV mass, stroke volume and cardiac output in WT CLP compared with sham mice (*Table* S3). LVIDd and LVIDs were reduced in WT CLP compared with sham mice. In contrast, LV mass, stroke volume, cardiac output and LVIDd remained unchanged in CLP compared with sham KO mice, whereas LVIDs was also reduced in septic KO mice. Septic WT mice showed a decrease in peak mitral inflow E and A and a reduced E/A ratio indicative for a decreased diastolic function. These parameters remained unaltered in CLP KO mice. Our data indicate that *Nlrp3* deletion protects from cardiac atrophy and systolic and diastolic dysfunction during sepsis.

Because NLRP3 mediates the activation of IL‐1β,^13^ we quantitated IL‐1β serum levels in WT and KO mice. At baseline, IL‐1β serum levels were reduced in KO compared with WT mice (*Figure* 1D). During sepsis, IL‐1β serum levels increased in WT mice, which was attenuated in KO mice (*Figure* 1D). In contrast, cardiac *Il1b* expression in sepsis (*Figure* 1E) was independent of the genotypes. Because IL‐1β induces interleukin 6 (*Il6)* expression in myocytes,^31^ we investigated if decreased IL‐1β serum levels were accompanied with a decreased cardiac *Il6* expression in septic *Nlrp3* mice. Indeed, cardiac *Il6* expression increased in septic WT but not KO mice (*Figure* 1F). These data indicate that *Nlrp3* deletion leads to decreased IL‐1β serum levels and an attenuated expression of IL‐1β‐target genes in the heart during sepsis.

In summary, our *in vivo* experiments revealed that decreased IL‐1β serum levels were paralleled by an attenuation of cardiac and cardiomyocyte atrophy and an improved diastolic as well as systolic cardiac function in septic *Nlrp3* KO mice when compared with septic WT mice. To investigate if these effects are mediated by IL‐1β, we tested if it reduces cardiomyocyte relaxation and contraction and mediates cardiomyocyte atrophy *in vitro*.

### IL1‐β leads to a reduction in cardiomyocyte contractility *in vitro*

**Figure 2:**
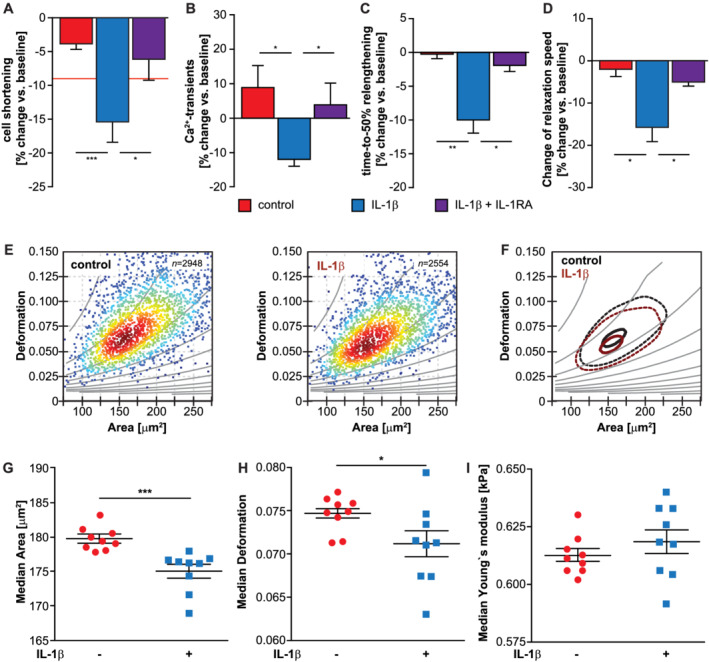
IL‐1β via the IL‐1 receptor reduces contractility, relaxation and deformation of cardiomyocytes. (A–D) Adult rat ventricular cardiomyocytes were treated with vehicle (PBS), recombinant IL‐1β (10 ng/mL) or the IL‐1 receptor antagonist (IL‐1RA) (10 μg/mL), as indicated, and IonOptix measurements were performed. Quantitation of effects on cell shortening *(A)* and Ca^2+^ transients *(B)* are shown. The threshold is defined as a reduction of cell shortening by −7.70% and is displayed by the red line. It equates to the mean effect on cell shortening induced by vehicle minus twice the standard deviation of this effect. *(C)* Time‐to‐50% relengthening. *(D)* Change of relaxation speed. Values are expressed as mean percentage change from baseline ± SEM. *P ≤ 0.05; **P ≤ 0.01; ^***^ *P* ≤ 0.001. (E–I) Mechanical high‐throughput characterization of HL‐1 cardiomyocytes by RT‐DC was used to investigate the effects of IL‐1β to cell size and deformation. *(E)* RT‐DC scatter plots of cell size (area, μm^2^) and deformation of vehicle (PBS, control, *n* = 2948 cells) and IL‐1β (n = 2554 cells) treated HL‐1 cells. Isoelasticity lines in grey highlight areas of equal elastic Young's modulus. Colour code indicates red (maximum) to blue (minimum) cell density. *(F)* Contour plot showing 50% (dashed) and 90% (solid) of maximum event density in vehicle (black) and IL‐1β (brown) treated HL‐1 cells. Statistical analysis comparing cells size (median area; *G*), median deformation *(H)* and Young's modulus *(I)* of experimental triplicates from three different days using linear mixed models. Error bars represent SEM of the distribution. *P ≤ 0.05; ^***^ *P* ≤ 0.001.

We next performed IonOptix measurements to investigate if IL‐1β affects cardiomyocyte contractility and relaxation. IL‐1β caused a significant reduction in cardiomyocyte shortening and Ca^2+^‐transients compared with vehicle (*Figure* 2A and 2B). IL‐1β also caused a significant increase in time‐to‐50%‐relengthening and a reduced relaxation speed compared with vehicle (*Figure* 2C and 2D). An IL‐1RA (*Figure* 2A–D) attenuated those effects, indicating that IL‐1β via its receptor decreases both cardiomyocyte contraction and relaxation.

To characterize the effects of IL‐1β on mechanical properties of cardiomyocytes, we performed RT‐DC measurements allowing single‐cell analyses in a high‐throughput fashion. We subjected IL‐1β and vehicle‐treated HL‐1 cardiomyocytes to RT‐DC and measured cell size and deformation. Compared with vehicle‐treated cells, IL‐1β‐treated cardiomyocytes were significantly smaller (*Figure* 2E–G) and showed a significantly reduced deformation (*Figure* 2E, 2F and 2H), but did not differ in their mechanical properties (*Figure* 2I). These data indicate that IL‐1β‐mediated atrophy is not compensated by structural modifications, leading to a change in cardiomyocyte stiffness.

### RNA sequencing analyses, autophagy and lysosomal protein degradation

**Figure 3:**
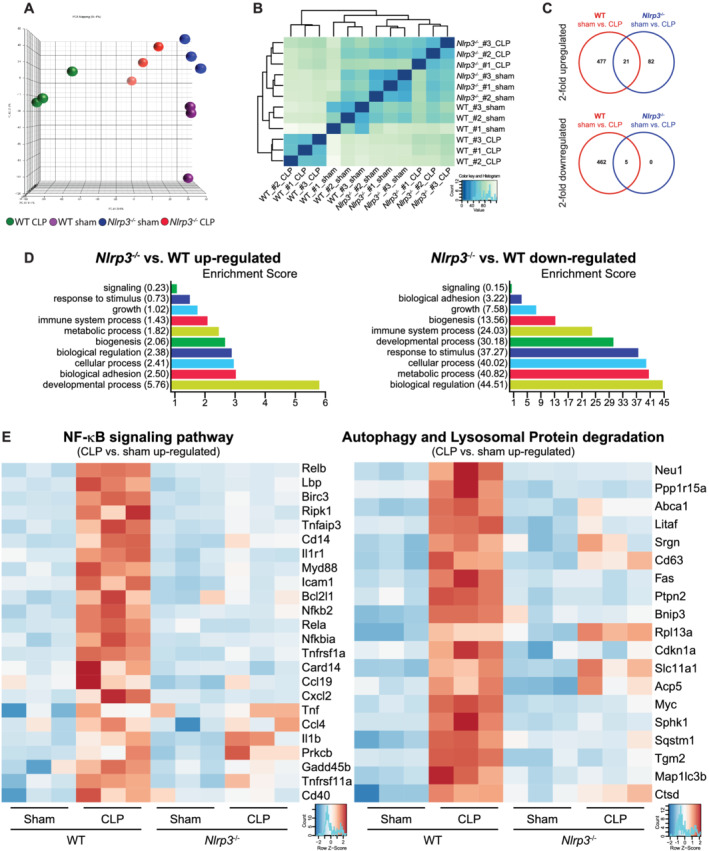
Principal component analysis and hierarchical clustering of RNA sequencing data. *(A)* Principal component analysis (PCA) performed using DESeq2 rlog‐normalized RNA‐seq data. Loadings for principal components 1 (PC1), PC2 and PC3 are shown in graph on *x*‐, *y*‐ and *z*‐axis. Genotypes and treatments are indicated. *(B)* Hierarchical clustering analyses performed using DESeq2 rlog‐normalized RNA‐seq data. Colour code (from white to dark blue) refers to the distance metric used for clustering (dark blue corresponds to the maximum of correlation values). Genotypes and treatments are indicated. *(C)* Venn diagrams showing the number of twofold upregulated (top panel) and twofold downregulated (bottom panel) genes and their overlap in hearts of septic *Nlrp3* WT and *Nlrp3* KO mice. *(D)* Selected enrichment scores of GO term analysis of genes, which were upregulated (left panel) or downregulated (right panel) in hearts of septic *Nlrp3* KO compared with *Nlrp3* WT mice. *(E)* Heatmaps of normalized expression values of genes involved in the NF‐κB signalling pathway (left panel) and the autophagy and lysosomal pathway of protein degradation (right panel). Genotypes and treatments as well as the *z*‐score are indicated.

To elucidate the mechanism involved in septic cardiomyopathy and cardiac atrophy *in vivo*, we performed next‐generation sequencing of RNAs isolated from hearts of CLP‐ and sham‐treated WT and KO mice. Total raw reads, mean read length, mapped reads and assigned reads for each sample analysed are shown in *Tables* S4 and S5. Principal component analysis (PCA) (*Figure* 3A) indicated a good separation between normalized read counts of all experimental groups. A homogeneous clustering and grouping were obtained using hierarchical clustering on the same data (*Figure* 3B). Comparison of the transcriptional profiles revealed that sepsis resulted in an upregulation of 498 genes in WT (*Table* S6) but only 103 genes in KO hearts (*Table* S7). From the upregulated genes, only 21 genes were increased in both septic WT and KO hearts (*Table* S8). Sepsis resulted in a downregulation of 467 genes in WT (*Table* S9) but only five genes in KO hearts (*Table* S10); those five genes were also decreased in hearts of septic WT mice (*Table* S11). These data indicate that *Nlrp3* deletion results in a blunted gene expression response in the heart during sepsis. Upregulated as well as the downregulated genes were annotated using the Kyoto Encyclopedia of Genes and Genomes (KEGG) database to identify enriched categories. Pathways that are likely influenced by DEG are shown as *Table* S12 (≥2‐fold upregulated in WT CLP vs. WT sham), *Table* S13 (≥2‐fold downregulated in WT CLP vs. WT sham) and *Table* S14 (≥2‐fold upregulated in KO CLP vs. KO sham). Due to the low number of downregulated genes in hearts of KO CLP vs. KO sham mice, no KEGG pathway could be identified. Importantly, genes upregulated in WT CLP‐ vs. WT sham‐treated mice belonged to the TNF signalling pathway, NF‐κB signalling pathway, insulin resistance, lysosome and the TGF‐ß signalling pathway (*Table* S12), which were all not detected in hearts of KO CLP vs. KO sham mice (*Table* S14). These data indicate that inflammation‐related pathways and pathways related to lysosome‐associated protein degradation are induced in hearts of septic WT but not KO mice. Gene Ontology (GO) term analysis revealed significant enrichment of genes belonging to adhesion and developmental processes in hearts of septic KO compared with WT mice (*Figure* 3D, left). Genes involved in biological regulation, metabolic and cellular process, developmental process and immune system process were decreased in hearts of septic KO when compared with WT mice (*Figure* 3D, right). Importantly, pronounced differences were found in the regulation of genes involved in the GO term ‘immune system process’ (*Figure* S2). Further, genes involved in NF‐κB signalling were increased in hearts of septic WT but not KO mice (*Figure* 3E, left). Among those 498 genes upregulated in hearts of septic WT mice 19 genes belonged to the autophagy and lysosomal pathway (ALP) of protein degradation (*Figure* 3E, right), whereas only one of these genes (*Rpl13a*, ribosomal protein L13a) was also increased in hearts of septic KO mice. In summary, these data indicate that deletion of *Nlrp3* is accompanied by less pronounced changes in overall gene expression, a reduced immune response, a reduction in NF‐κB signalling and increased ALP in the heart during sepsis.

**Figure 4:**
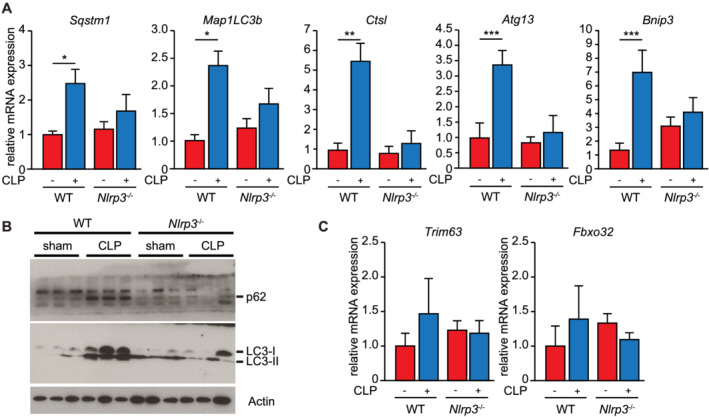
Sepsis‐induced expression of autophagy genes is attenuated in hearts of *Nlrp3* KO mice. *(A)* qRT‐PCR analysis of *Sqstm1*, *Map 1LC3b*, *Ctsl*, *Atg13* and *Bnip3* in hearts of *Nlrp3* WT sham (*n* = 13), *Nlrp3* WT CLP (*n* = 12), *Nlrp3* KO sham (*n* = 8) and *Nlrp3* KO CLP (*n* = 15) mice. mRNA expression was normalized to *Gapdh*. *(B)* Western blot analysis with anti‐p62 and anti‐LC3B antibody. Actin was used as loading control. *(C)* qRT‐PCR analysis of *Trim63* and *Fbxo32* in hearts of *Nlrp3* WT sham, *Nlrp3* WT CLP, *Nlrp3* KO sham and *Nlrp3* KO CLP mice. mRNA expression was normalized to *Gapdh*. Data are presented as mean ± SEM. *P ≤ 0.05; **P ≤ 0.01; ^***^ *P* ≤ 0.001.

To confirm changes in ALP gene expression, we performed qPCR and Western blot analyses. We found an increase in *Sqstm1* (encoding p62) and *Map 1LC3b* (encoding LC3) expression in hearts of septic WT but not KO mice (*Figure* 4A). P62 as well as LC3‐II protein contents were increased in hearts of septic WT but not KO mice (*Figure* 4B). We also found an increase in autophagy‐related protein 13 (*Atg13*), cathepsin L (*Ctsl*) and Bcl2 interacting protein 3 (*Bnip3*), expression in hearts of septic WT but not KO mice (*Figure* 4A). Because the ubiquitin–proteasome system is involved in muscle atrophy and mediates the breakdown of structural proteins, especially myosin heavy chain,^38^ we investigated the regulation of its key factors *Fbxo32*/atrogin1 and *Trim63*/MuRF1 but did not find them to be regulated (*Figure* 4C). These data indicate that NLRP3/IL‐1β contributes to an increased cardiac ALP gene expression in septic mice.

### IL‐1β‐induced cardiomyocyte atrophy and influenced autophagy genes *in vitro*

**Figure 5:**
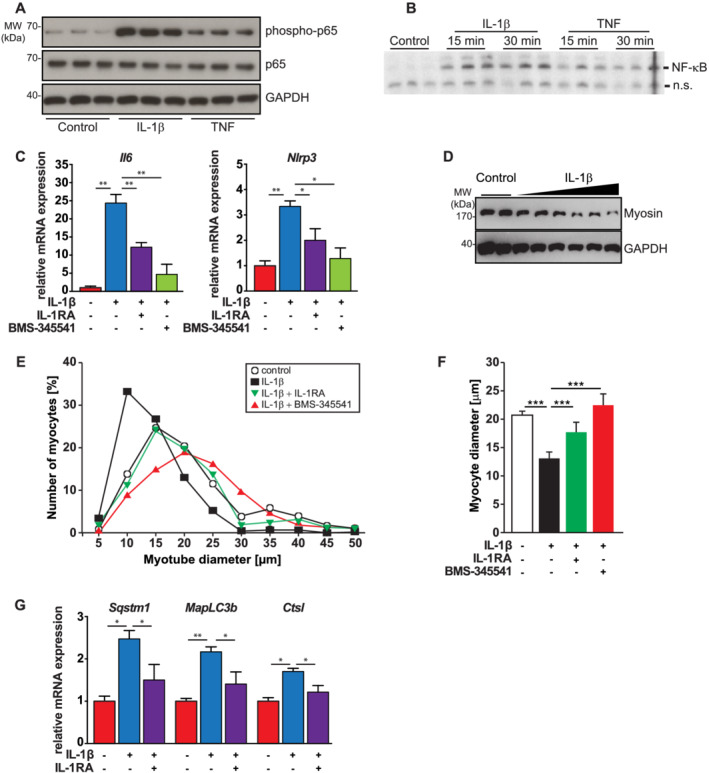
The IL‐1β signalling pathway is contained and active in H9c2 cardiomyocytes. *(A)* Seven days differentiated H9c2 myotubes were treated with recombinant vehicle, IL‐1β (50 ng/mL) and TNF (5 ng/mL), as indicated, for 25 min. Western blot analysis was performed with anti‐phospho‐NF‐κB p65 and anti‐NF‐κB p65 antibody. GAPDH was used as loading control. *(B)* Differentiated H9c2 cells were treated with recombinant vehicle, IL‐1β (50 ng/mL) and TNF (5 ng/mL), as indicated, for 15 and 35 min, and NF‐κB‐DNA‐response element complex formation was analysed by EMSA. *(C)* Differentiated H9c2 cells were treated with recombinant IL‐1β (50 ng/mL), vehicle, the IL‐1 receptor antagonist (IL‐1RA) (10 μg/mL) and the NF‐κB inhibitor BMS‐345541 (25 μM), as indicated. qRT‐PCR analysis of *Il6* (left panel) and *Nlrp3* (right panel) is shown. mRNA expression was normalized to *Gapdh*. *(D)* Differentiated H9c2 cells were treated with solvent or increasing amounts of recombinant IL‐1β (10, 20 and 50 ng/mL) for 72 h. Western blot analysis of isolated proteins with anti‐myosin heavy chain (MyHC)‐slow antibody is shown. GAPDH was used as loading control. *(E)* Frequency distribution histograms of cell width of vehicle, IL‐1β (50 ng/mL), IL‐1RA (10 μg/mL) and BMS‐345541 (25 μM)‐treated myotubes, as indicated, are shown, *n* = 100 cells per condition. *(F)* Mean myotube width. *(G)* Differentiated H9c2 cells were treated with vehicle or recombinant IL‐1β (50 ng/mL), as indicated, for 72 h. qRT‐PCR analysis of *Sqstm1*, *MapLC3b* and *Ctsl* is shown. mRNA expression was normalized to *Gapdh*. Data are presented as mean ± SEM. *P ≤ 0.05; **P ≤ 0.01; ^***^ *P* ≤ 0.001.

Because IL‐1β activates NF‐κB signalling in skeletal myocytes^31^ and lymphocytes, and our RNA sequencing data revealed an activated NF‐κB pathway in septic WT but not KO hearts, which was paralleled by a reduction in IL‐1β serum levels in septic KO mice, we investigated if IL‐1β stimulates the NF‐κB pathway in cardiomyocytes. Western blot analysis showed that IL‐1β increased NF‐κB p65 phosphorylation in H9c2 cells (*Figure* 5A). Electrophoretic mobility shift assay (EMSA) revealed that IL‐1β caused an increased binding of activated NF‐κB p65 to NF‐κB response elements (*Figure* 5B). qRT‐PCR analysis showed that IL‐1β increased the expression of its target genes *Il6* and *Nlrp3* in myocytes, which was attenuated by IL‐1RA and the IKKβ‐directed NF‐κB inhibitor BMS‐345541, respectively (*Figure* 5C). These data indicate that IL‐1β, via the IL‐1 receptor, activated the NF‐κB p65 pathway, which increased the expression of IL‐1β target genes in cardiomyocytes.

To analyse if IL‐1β causes myocyte atrophy, we treated H9c2 myotubes with increasing amounts of IL‐1β and vehicle for 72 h and measured myotube diameters. IL‐1β treatment resulted in a dose‐dependent decrease in myotube diameters of 25%, 35% and 40%, respectively (*Figure* S3A–C). IL‐1β treatment led to a dose‐ and time‐dependent decrease in myosin heavy chain (MyHC) protein content in H9c2 cells, which is consistently observed in atrophy^31^, ^38^ (*Figures* 5D and S3D). IL‐1RA (*Figure* 5E and 5F) and BMS‐345541 (*Figure* 5E and 5F) attenuated IL‐1β‐induced atrophy, indicating that the IL‐1 receptor and the IKK/NF‐κB pathway were involved in this phenotype.

Based on our RNA sequencing data, we next hypothesized that IL‐1β increases ALP gene expression. Using qPCR, we found that IL‐1β treatment increased *Sqstm1*/p62, *Map 1lc3b*/LC3b and *Ctsl* expression in H9c2 myotubes, which was attenuated by IL‐1RA (*Figure* 5G). In contrast, IL‐1β treatment had no effect on *Fbxo32*/atrogin1 and *Trim63*/MuRF1 expression (data not shown). These data indicate that IL‐1β‐induced cardiomyocyte atrophy is likely mediated by ALP‐dependent protein degradation and that MuRF1 and atrogin1 are possibly not involved in this process.

## Discussion

Sepsis is a life‐threatening organ dysfunction caused by a dysregulated host response to an infection. It is frequently accompanied by septic cardiomyopathy, which increases mortality. During sepsis, the cardiodepressant and inflammatory cytokine IL‐1β rapidly increases.^7^ However, the role of IL‐1β in septic cardiomyopathy is not well understood. Here, we present evidence that septic cardiomyopathy concerns both systolic and diastolic function. We show that inhibition of the NLRP3/IL‐1β axis protects against systolic and diastolic dysfunction in sepsis. We used *Nlrp3*‐deficient mice to investigate if activation of IL‐1β is involved in septic cardiomyopathy. *Nlrp3*‐deficient mice showed an improved systolic and diastolic cardiac function and increased survival in sepsis. Our results are in accordance with recently published work showing that *Nlrp3* deletion reduces mortality^31^ and sepsis‐induced end‐organ damage, such as cardiomyopathy,^15^ acute lung injury and muscle failure.^31^ Pharmacological inhibition of NLRP3 might therefore be beneficial in sepsis. Indeed, positive effects were reported for the NLRP3 inhibitors hemin, which protected against CLP‐induced acute lung injury in mice,^39^ and scutellarin^40^ and glyburide,^41^ which improved survival of mice with bacterial sepsis. If such an approach is applicable to critically ill human patients awaits further investigation.

Because cardiac function often normalizes in human patients after sepsis,^6^ functional rather than structural abnormalities are thought to cause septic cardiomyopathy.^42^ However, structural changes in the heart were also reported.^12^ Here, we show that cardiac function and structure deteriorates during sepsis and that inhibition of NLRP3/IL‐1β improves both entities. Early clinical studies identified IL‐1β as a myocardial depressant factor that mediates HF in sepsis.^11^ Elevated IL‐1β serum levels are found in septic human patients and animal models.^7^, ^43^ In *Nlrp3*‐deficient mice, we found reduced IL‐1β serum levels, both at baseline and after CLP, reflecting the canonical pathway of proteolytic cleavage of pro‐IL‐1β to IL‐1β by the NLRP3/caspase 1 pathway.^13^ Nevertheless, residual IL‐1β amounts in serum of *Nlrp3* KO mice implicate additional pathways involved in IL‐1β‐activation. In fact, a non‐canonical pathway involving caspase 11 (in human caspase 4/5)^44^ and an alternative pathway involving caspase 8^45^ also activate IL‐1β and may account for residual IL‐1β levels. Although our data suggest that the NLRP3 pathway primarily activates IL‐1β in sepsis, further studies are needed to elucidate the role of the non‐canonical and alternative IL‐1β‐activating pathway in sepsis. A recent study reported that the complement system activates the cardiac NLRP3 inflammasome in CLP‐induced sepsis mediating cardiomyopathy and *Nlrp3* deletion was shown to be cardioprotective,^15^ which is in line with our data. In addition to this study, we attribute the beneficial effects seen in KO mice to effects of IL‐1β on cardiomyocytes. This is supported by previous work showing that IL‐1β decreases myocardial contractility *in vitro* and *ex vivo* ^9^, ^10^ possibly explaining the preserved cardiac function seen in septic *Nlrp3* KO mice that have reduced IL‐1β serum levels. Also, depletion of IL‐1β by immunoadsorption reduced the NIA of serum from septic human patients on cardiomyocytes.^11^ Likewise, the haemodynamic and metabolic manifestations of septic shock were attenuated by an IL‐1β antagonist.^8^ We show that IL‐1β, via the IL‐1 receptor, negatively affects contraction and relaxation of cardiomyocytes and reduces Ca^2+^ transients. The latter agrees with previous reports showing that IL‐1β negatively affects Ca^2+^ signalling in cardiomyocytes causing proarrhythmic effects.^46^ Therefore, decreased IL‐1β levels might have contributed to cardioprotection in septic KO mice. Our data also implicate that IL‐1β contributes to diastolic cardiac dysfunction during sepsis. First, IL‐1β via its receptor prolonged cardiomyocyte relaxation and reduced deformation. Second, diastolic cardiac function was preserved in *Nlrp3* KO mice during sepsis. Overall, we show that IL‐1β is involved in the pathogenesis of systolic and diastolic dysfunction by decreasing cardiomyocyte contractility and relaxation and that inhibition of IL‐1β could have beneficial effects in sepsis. Our data extend the findings of Kalbitz et al.,^15^ who showed a trend towards a better diastolic function in *Nlrp3* KO mice compared with controls, which did not reach statistical significance 8 h after CLP surgery. Because we performed echocardiography 24 h after surgery, we hypothesize that this discrepancy is related to differences in timing. It is also tempting to speculate that IL‐1β‐induced cardiac atrophy facilitates diastolic cardiac function, which in turn occurs at later time points during sepsis. However, if IL‐1β directly mediated cardiomyopathy and cardiac atrophy in sepsis needs to be proven. To this end, we assume that administration of IL‐1β will reverse the cardioprotective effects seen in septic *Nlrp3* KO mice.

Whether or not decreased cardiac function in sepsis is a consequence of structural changes is not well understood. Here, we show that IL‐1β via its receptor and NF‐κB causes cardiomyocyte atrophy, which was accompanied by a reduced MyHC content, and that KO mice were protected from sepsis‐induced cardiac atrophy. This observation coincided with an improved cardiac function in septic KO mice indicative for a close structure–function relationship. RT‐DC measurements revealed an increased stiffness of cardiomyocytes in response to IL‐1β, further implicating that IL‐1β causes structural changes. Further, IL‐1β‐induced structural changes are manifested as cardiac and cardiomyocyte atrophy with a decrease in contractile sarcomeric proteins negatively affecting cardiac function. We hypothesize that the preserved cardiac function in septic KO mice was related to both functional and structural effects of IL‐1β on the heart.

Recently, we showed that IL‐1β causes muscle atrophy and reported an increase in atrogin1/*Fbxo32* and MuRF1/*Trim63* expression in skeletal muscle of septic mice.^31^ However, both genes remained unchanged in hearts of septic mice, and IL‐1β had no effect on their expression in cardiomyocytes. Therefore, we hypothesize that ALP‐dependent protein degradation may be involved in sepsis‐induced cardiac atrophy. Indeed, ALP genes were enriched in hearts of septic WT but not KO mice, indicating that two distinct protein‐degrading pathways mediate heart and skeletal muscle atrophy in sepsis. We also found an increase in LC3‐II and p62 proteins in hearts of septic WT but not KO mice. *In vitro* experiments showed that IL‐1β via its receptor contributes to transcriptional regulation of selected ALP genes, suggesting that it is involved in sepsis‐induced cardiac atrophy. Indeed, clinical and preclinical studies indicate that sepsis and ALP are closely related in multiple organs including the heart.^47^, ^48^, ^49^ However, the role of ALP in sepsis‐related end‐organ damage is not well understood. On one hand, ALP inhibition was shown to improve cardiac function in lipopolysaccharide (LPS)‐treated mice, indicating that ALP is maladaptive,^49^ which is in line with our data. On the other hand and in contrast to our data, *in vivo* models using CLP‐induced sepsis^47^ and *in vitro* models using LPS‐treated cardiomyocytes suggest that ALP activation is cardioprotective. Therefore, further studies are needed to elucidate the role of ALP in septic cardiomyopathy.

Previously, it was shown that an activated NLPR3 inflammasome in the heart mediates LPS‐induced cardiac dysfunction, inflammation, apoptosis and pyroptosis *in vivo* and *in vitro*.^16^ Although the LPS and the CLP model are not directly comparable, these data support cardioprotective effects of NLRP3 inhibition. In addition, the NLRP3 inflammasome in cardiomyocytes was shown to be involved in pressure overload‐induced HF.^22^ Although this mechanism might account for the cardioprotective effects seen here, we did not investigate the role of NLRP3 in cardiomyocytes, but focused on the effects of IL‐1β. Finally, the NLRP3 inflammasome also mediates the conversion of pro‐IL‐18 to the cardioactive cytokine IL‐18.^13^ IL‐18 mediates cardiac dysfunction in animal models of acute myocardial infarction and pressure overload, and its inhibition protects against LPS‐induced myocardial dysfunction. Although reduced IL‐18 levels might have contributed to the cardioprotective effects observed here, this hypothesis needs to be proven.

## Conclusions

**Figure 6:**
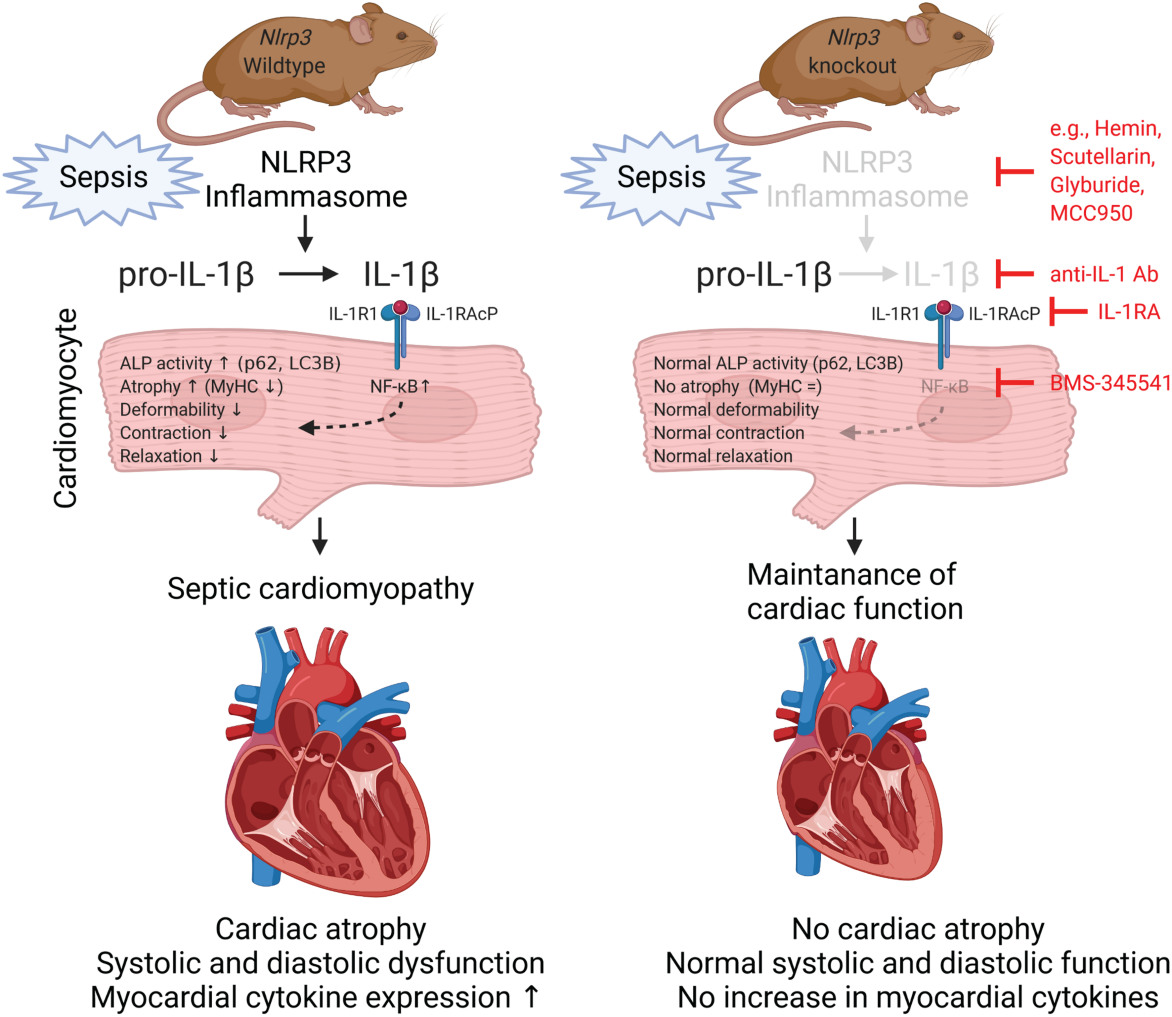
Proposed mechanism of septic cardiomyopathy. (Left panel) Sepsis, caused by polymicrobial infection leads to an activation of the NLRP3 inflammasome, resulting in increased conversion of inactive pro‐IL‐1ß in active IL‐1ß, which acts on cardiomyocytes via the IL‐1 receptor complex (IL‐1R1/IL‐1RAcP). Binding of IL‐1 to its receptor causes an activation of NF‐κB, which leads to increased protein degradation by the autophagy–lysosomal pathway (ALP), a decrease in myosin heavy chain content (MyHC), a reduced deformability and a decreased cardiomyocyte contraction and relaxation. This in turn causes septic cardiomyopathy with cardiac atrophy, systolic and diastolic cardiac dysfunction and increased expression of pro‐inflammatory cytokines in the heart. (Right panel) In *Nlrp3* knockout mice, sepsis can no longer activate the NLRP3 inflammasome, generation of IL‐1ß is much reduced, and activation of NF‐κB as well as NF‐κB signalling is attenuated, which maintains cardiomyocyte size, deformability, contraction and relaxation. As a result, *Nlrp3* knockout mice show a normal cardiac function in sepsis without cardiac atrophy and preserved expression of pro‐inflammatory cytokines. Inhibitors of the NLRP3 inflammasome (e.g. haemin, scutellarin, glyburide and MCC950), IL‐1ß (anti‐IL‐1 antibody; anti‐IL‐1 Ab), IL‐1 receptor antagonist (IL‐1 RA) and NF‐κB (IKKβ‐directed NF‐κB inhibitor BMS‐345541) that hold therapeutic potential are indicated.

NLRP3‐mediated IL‐1β activation in sepsis plays a role in in the pathogenesis of septic cardiomyopathy, leading to cardiac atrophy and decreased systolic and diastolic cardiac function (*Figure* 6). Polymicrobial sepsis leads to an activation of the NLRP3 inflammasome resulting in increased conversion of inactive pro‐IL‐1ß in active IL‐1ß, which acts on cardiomyocytes via the IL‐1 receptor complex (IL‐1R1/IL‐1RAcP). IL‐1 via its receptor activates NF‐κB, which causes an increased protein degradation by the ALP, a decrease in myosin heavy chain content (MyHC), a reduced deformability and a decreased cardiomyocyte contraction and relaxation. This in turn results in septic cardiomyopathy with cardiac atrophy, systolic and diastolic cardiac dysfunction and increased expression of pro‐inflammatory cytokines in the heart. In *Nlrp3* knockout mice, sepsis can no longer activate the NLRP3 inflammasome, generation of IL‐1ß is much reduced, and activation of NF‐κB as well as NF‐κB signalling is attenuated, which maintains cardiomyocyte size, deformability, contraction and relaxation. As a result, *Nlrp3* KO mice show a normal cardiac function in sepsis without cardiac atrophy and preserved expression of pro‐inflammatory cytokines. Inhibitors of the NLRP3 inflammasome (e.g. haemin, scutellarin, glyburide and MCC950), IL‐1ß (anti‐IL‐1 antibody; anti‐IL‐1 Ab), IL‐1 RA and NF‐κB (IKKβ‐directed NF‐κB inhibitor BMS‐345541), which are indicated, could be useful to prevent septic cardiomyopathy.

## Limitations

Sepsis in humans is a clinical problem that frequently occurs at the extremes of ages, especially in elderly patients. For our experiments, we have used 12‐ to 16‐week‐old mice, which relates to a young age in human patients. A comparable age range has also been used by other groups that investigated the role of the NLRP3 inflammasome in septic cardiomyopathy.^15^, ^24^, ^25^, ^28^, ^29^ Although the currently available data on the role of NLRP3 on septic cardiomyopathy are informative for a younger age, further studies on mature (12‐month‐old) and aged mice (24‐month‐old) are needed to provide data that are more translatable to aged patients with sepsis.

All animal experiments shown here have been performed in male mice. A gender imbalance is also apparent in work published by other groups that only investigated male mice,^15^, ^24^, ^25^ male rats^27^, ^30^ or female mice.^28^, ^29^ Because we cannot extrapolate our findings to female mice or even compare the effects between male and female mice, further sex‐specific studies are needed.

Given the role of the NLRP3 inflammasome in the heart,^15^, ^22^, ^26^, ^50^ it is tempting to speculate that its deletion in cardiomyocytes contributed to the attenuation of septic cardiomyopathy observed in *Nlrp3* KO mice. However, this question cannot be answered by our work. Based on our data, we hypothesize that reduced IL‐1β serum levels account for diminished cardiac atrophy and the improved cardiac function in septic *Nlrp3* KO mice. Further studies are needed to elucidate the contribution of the NLRP3 inflammasome contained in cardiomyocytes for the pathogenesis of septic cardiomyopathy, such as using cardiomyocyte‐specific *Nlrp3* KO mice.

## Declarations

Ethics approval and consent to participate.

The Landesamt für Gesundheit und Soziales, Berlin, Germany (G207/13), approved the animal studies (NIH publication No. 86–23, revised 1985).

## Funding

This work was supported by the Deutsche Forschungsgemeinschaft (DFG, German Research Foundation) (FI 965/5‐1, FI 965/5‐2 and FI 965/9‐1 to [JF] and 231396381/GRK1947 to [OO]), the DZHK (German Center for Cardiovascular Research), Partner Site Greifswald (81Z5400153 to [JF] and 81X3400107 to [OO]) and the Bundesministerium für Bildung und Forschung (ZIK HIKE) (03Z22CN11 to [OO]). OO is co‐founder and shareholder of Zellmechanik Dresden commercializing real‐time deformability cytometry.

## Conflict of interest

K.B._,_ M.K., N.H., T.E.K., M.S., A.Ha., S.G., M.T., S.S., B.C., M.W., St.G., D.B., A.He., C.S., C.B., S.B.F., F.C.L. and H.S. declare that they have no conflict of interest. The remaining authors have disclosed that they do not have any conflicts of interest.

## Supporting information


**Figure S1.** **Nlrp3 KO mice are protected from septic cardiomyopathy.** 12–16‐week‐old male Nlrp3 KO and WT mice were subjected to CLP or sham surgery. Haematoxylin and eosin (**A, B**) and trichrome (**C, D**) staining of histological sections from hearts of sham and CLP operated WT and Nlrp3 KO mice at 96 hours after surgery, as indicated, are shown. Gross morphology (**A, C**; Scale bar = 1 mm) and higher magnifications (**B, D**; Scale bar = 100 μm) are shown

**Figure S2.** **Genes involved in “Immune System Process” are less activated in hearts of septic Nlrp3 KO mice.** Heatmap of normalized expression values of genes involved in Immune System Process. Genotypes and treatments as well as the z‐score are indicated

**Figure S3.** **IL‐1β causes atrophy of cardiomyocytes in a dose‐ and time‐dependent manner.** Differentiated H9c2 myotubes were treated with increasing amounts of recombinant IL‐1β (10 ng/ml, 20 ng/ml, 50 ng/ml) or vehicle for 72 hours. **A**, Representative light microscopy pictures. Scale bar = 250 μm. **B**, Frequency distribution histograms of cell width of vehicle and IL‐1β (10 ng/ml, 20 ng/ml and 50 ng/ml) treated myotubes, n = 100 cells per condition. **C**, Mean myotube width. **D**, Differentiated H9c2 myotubes were treated with recombinant IL‐1β (50 ng/ml) or vehicle for 3 hours, 6 hours, 12 hours, 24 hours, 48 hours and 72 hours, respectively. Western blot analysis of isolated proteins using anti‐MyHC slow is shown. GAPDH was used as loading control. Data are presented as mean ± SEM. **p ≤ 0.01, ***p ≤ 0.001, ****p ≤ 0.0001

**Table S1.** Primer pairs for genotyping of Nlrp3 KO and Nlrp3 WT mice
Table S2. Body and organ weights 96 hours after CLP or sham surgery
Table S3. Data of transthoracic echocardiography
**Table S4.** Total raw reads, mean read length, mapped reads for each sample analysed
**Table S5.** Successfully assigned reads for each sample analysed

**Table S6.** ≥ 2‐fold upregulated genes in hearts of CLP vs. sham treated *Nlrp3* WT mice (.xls table; Table S5_wt_CLP vs wt_sham_FC2.xlsx)

**Table S7.** ≥ 2‐fold upregulated genes in hearts of CLP vs. sham treated *Nlrp3* KO mice (.xls table; Table S6_ko_CLP vs ko_sham_FC2.xlsx)
**Table S8.** Twentyone genes upregulated in both septic Nlrp3 WT and septic Nlrp3 KO hearts compared to the respective sham group

**Table S9.** ≥ 2‐fold downregulated genes in hearts of CLP vs. sham treated *Nlrp3* WT mice (.xls table; Table S8_wt_CLP vs wt_sham_FC‐2.xlsx)

**Table S10.** ≥ 2‐fold downregulated genes in hearts of CLP vs. sham treated *Nlrp3* KO mice (.xls table; Table S9_ko_CLP vs ko_sham_FC‐2.xlsx)
**Table S11.** Five genes downregulated in both septic Nlrp3 WT and septic Nlrp3 KO hearts compared to the respective sham group
Table S12. KEGG pathway analysis of significantly upregulated genes in septic Nlrp3 WT compared to sham treated Nlrp3 WT hearts
Table S13. KEGG pathway analysis of significantly downregulated genes in septic Nlrp3 WT compared to sham treated Nlrp3 WT hearts
Table S14. KEGG pathway analysis of significantly upregulated genes in septic Nlrp3 KO compared to sham treated Nlrp3 KO hearts
**Table S15.** Primer pairs for quantitative real‐time‐PCR are shown
