# Supplementary figures and images for: Inhibition of the NLRP3/IL‐1β axis protects against sepsis‐induced cardiomyopathy
Source: J Cachexia Sarcopenia Muscle. 2021 Sep 2;12(6):1653–68. doi: 10.1002/jcsm.12763 (PMC8718055; doi:10.1002/jcsm.12763)

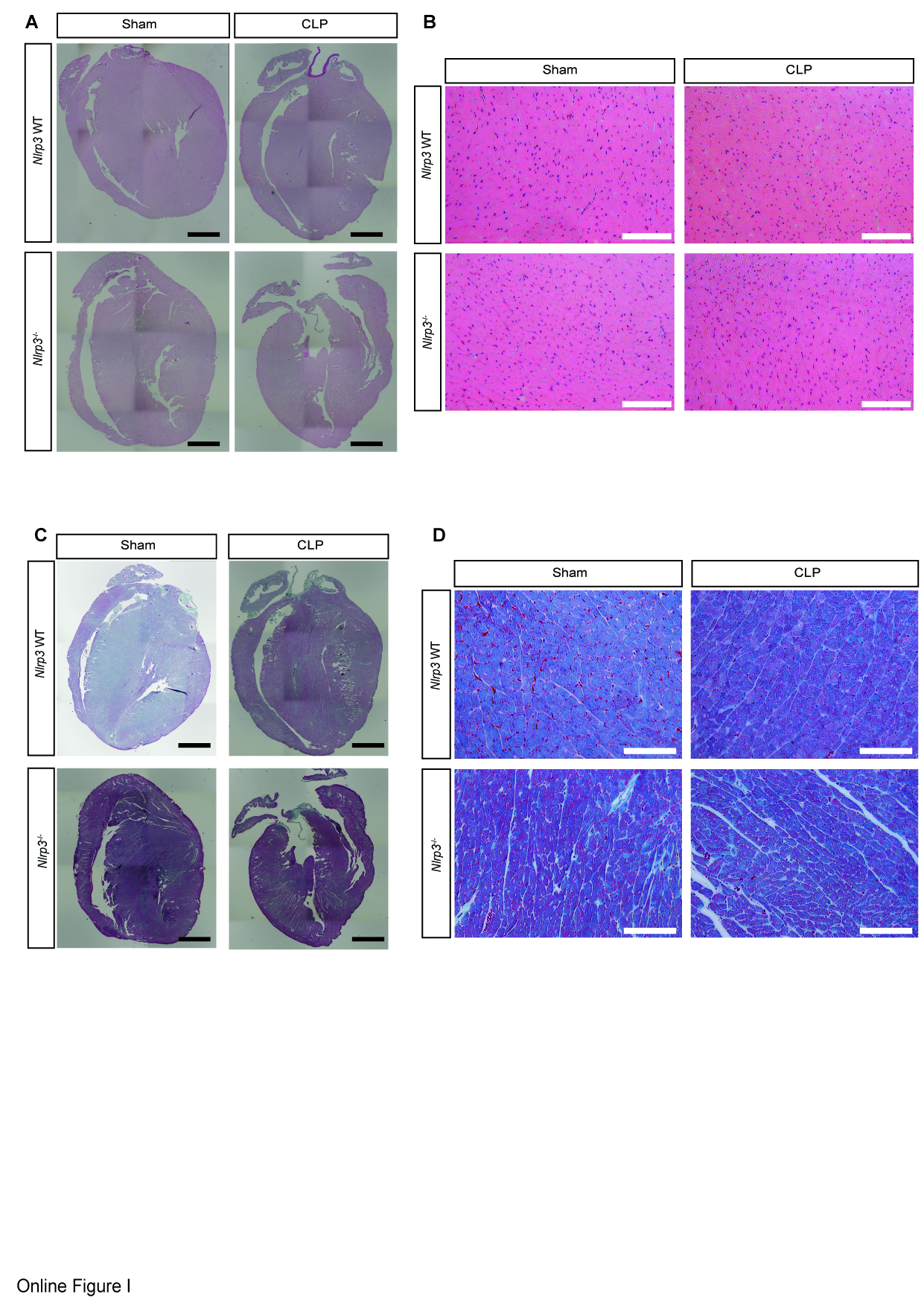

Supplement: Supplementary file 1 — Figure S1. Nlrp3 KO mice are protected from septic cardiomyopathy. 12–16‐week‐old male Nlrp3 KO and WT mice were subjected to CLP or sham surgery. Haematoxylin and eosin (A, B) and trichrome (C, D) staining of histological sections from hearts of sham and CLP operated WT and Nlrp3 KO mice at 96 hours after surgery, as indicated, are shown. Gross morphology (A, C; Scale bar = 1 mm) and higher magnifications (B, D; Scale bar = 100 μm) are shown [file JCSM-12-1653-s002.tif]

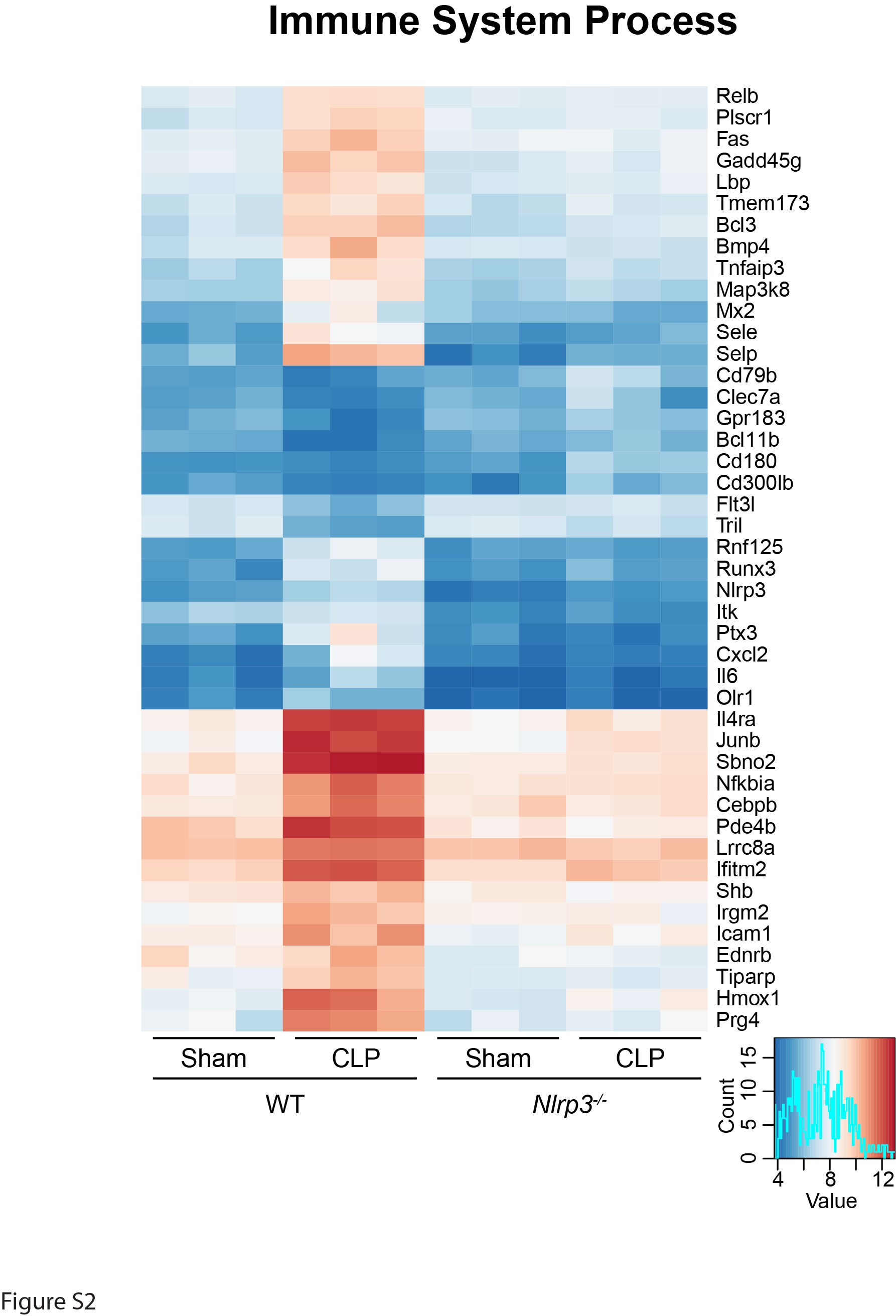

Supplement: Supplementary file 2 — Figure S2. Genes involved in “Immune System Process” are less activated in hearts of septic Nlrp3 KO mice. Heatmap of normalized expression values of genes involved in Immune System Process. Genotypes and treatments as well as the z‐score are indicated [file JCSM-12-1653-s001.jpg]

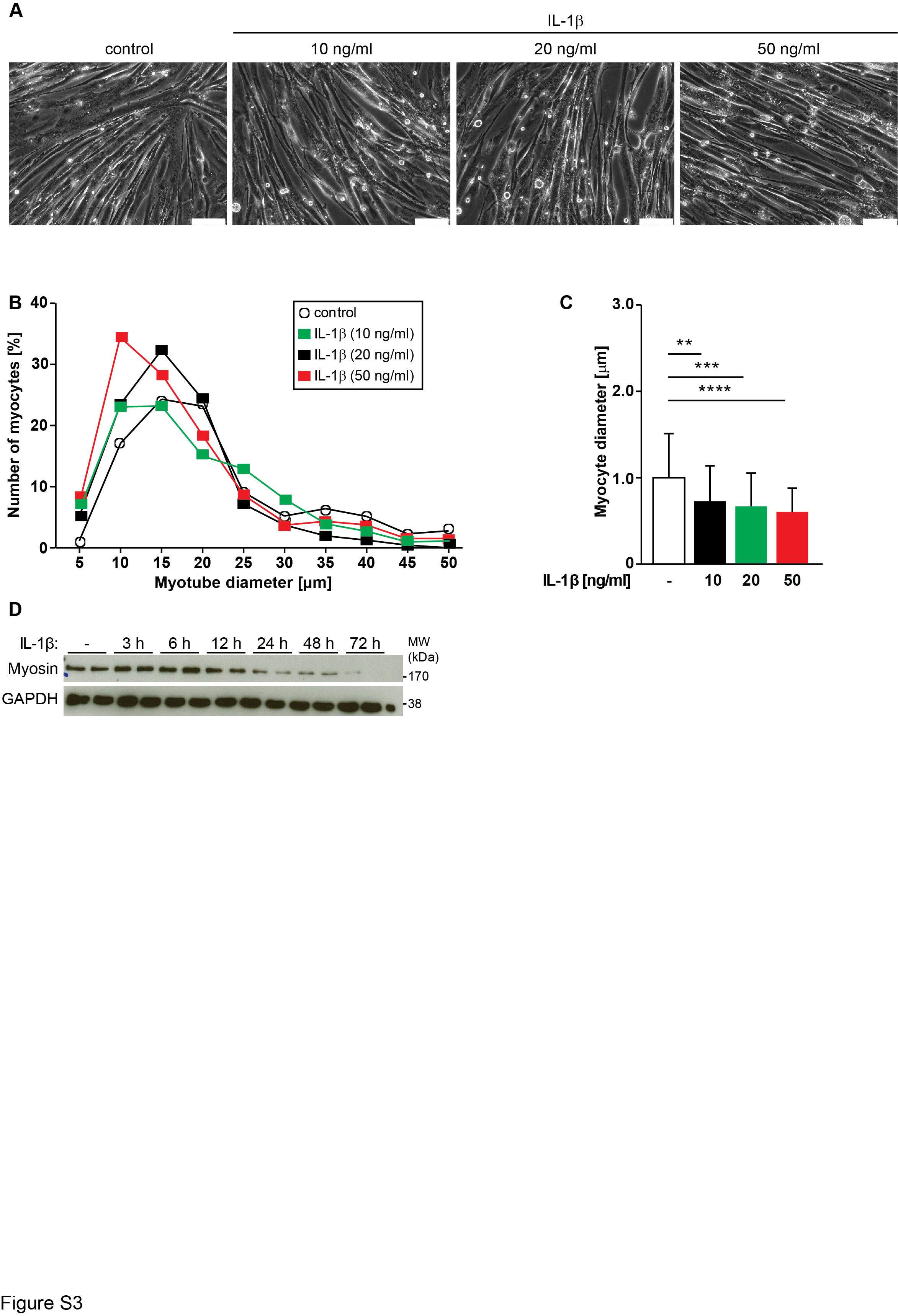

Supplement: Supplementary file 3 — Figure S3. IL‐1β causes atrophy of cardiomyocytes in a dose‐ and time‐dependent manner. Differentiated H9c2 myotubes were treated with increasing amounts of recombinant IL‐1β (10 ng/ml, 20 ng/ml, 50 ng/ml) or vehicle for 72 hours. A, Representative light microscopy pictures. Scale bar = 250 μm. B, Frequency distribution histograms of cell width of vehicle and IL‐1β (10 ng/ml, 20 ng/ml and 50 ng/ml) treated myotubes, n = 100 cells per condition. C, Mean myotube width. D, Differentiated H9c2 myotubes were treated with recombinant IL‐1β (50 ng/ml) or vehicle for 3 hours, 6 hours, 12 hours, 24 hours, 48 hours and 72 hours, respectively. Western blot analysis of isolated proteins using anti‐MyHC slow is shown. GAPDH was used as loading control. Data are presented as mean ± SEM. **p ≤ 0.01, ***p ≤ 0.001, ****p ≤ 0.0001 [file JCSM-12-1653-s004.jpg]
