# Supplementary material for: Inhibition of the NLRP3/IL‐1β axis protects against sepsis‐induced cardiomyopathy
Source: J Cachexia Sarcopenia Muscle. 2021 Sep 2;12(6):1653–68. doi: 10.1002/jcsm.12763 (PMC8718055; doi:10.1002/jcsm.12763)
Supplement: Supplementary file 4 — Table S1. Primer pairs for genotyping of Nlrp3 KO and Nlrp3 WT mice Table S2. Body and organ weights 96 hours after CLP or sham surgery Table S3. Data of transthoracic echocardiography Table S4. Total raw reads, mean read length, mapped reads for each sample analysed Table S5. Successfully assigned reads for each sample analysed [file JCSM-12-1653-s008.docx]

**Supporting Information**

**Supporting Materials & Methods**

**Histological and immunohistochemical analyses.**

96 hours after sham and CLP surgery mice were sacrificed by cervical dislocation and hearts, lungs and livers were harvested. Body weight was measured before and after the experiment. Organ weights were related to tibia length. Hearts were either flash frozen in gum tragacanth (Sigma-Aldrich) or fixed in 4 % paraformaldehyde, and processed for routine histology as described previously.[1] Frozen sections were cut on a cryotome (Leica CM 3050 S, Leica Microsystems GmbH, Germany) and stained with haematoxylin-eosin or Masson’s trichrome as previously described.[2] Images were acquired with a Leica CTR 6500 HS microscope, and Leica digital camera DFC 425 (Leica Microsystems GmbH, Germany). Myocyte cross sectional area (MCSA) of cardiomyocytes were measured from hematoxylin and eosin-stained histological cross sections from hearts (n=100 myocytes per mouse). The person who performed MCSA measurements was blinded to the genotype and condition the animal was exposed to during the experiment.

**Measurements of serum IL-1β**

Measurements of serum IL-1β were performed by using the Mouse ELISA Kit for IL-1β (Abcam, ab100704) according to the manufacturers’ protocol.

**HL-1 cell culture**

HL-1 cells were cultured, under sterile conditions in Claycomb media (Sigma-Aldrich) supplemented with norepinephrine (100 μM, Sigma-Aldrich), fetal bovine serum (FBS, 10%, Sigma-Aldrich), L-glutamine (2 mM, Sigma, UK), penicillin (100 IU/ml) and streptomycin (100 µg/ml, Sigma-Aldrich) as described in the literature.[3] Cells were incubated at 37 ºC, 95 % O_2_, 5 % CO_2_, 90 % humidity on fibronectin-coated culture flasks for maintenance and fibronectin-coated 6-well plates (Falcon™, Fisher Scientific) for cytokine treatments and RT-DC measurements. HL-1 cells were treated with recombinant IL-1β (GIBCO®, Invitrogen) or vehicle (PBS) for 24 hours. An IL-1RA (10 µg/ml, Sigma-Aldrich) was added 1 hour prior to IL-1β treatment.

**RNA sequencing and statistical analyses**

For all sample types (*Nlrp3* WT sham, *Nlrp3* WT CLP, *Nlrp3* KO sham, *Nlrp3* KO CLP) three biological replicates were sequenced using the IonTorrent proton^TM^ sequencing technology. In total, three sequencing runs were accomplished each multiplexed with one replicate of each sample type. The raw sequence data in FASTQ-format are stored in the Sequence Read Archive (SRA) at National Center for Biotechnology Information (NCBI) under the accession number SRP00123 (https://www.ncbi.nlm.nih.gov/). The sequence quality of each FASTQ-file was analysed by applying FastQC (version 0.11.1).[4] Thereafter, using FastQC all remaining sequencing adapters were trimmed and all reads were trimmed with regard to their sequencing quality (phred score<20). The trimmed short reads for each sample were aligned onto the mouse mm10 genome by applying the Torrent Mapping Alignment Program (TMAP aligner) (version 2.9.2) specifically developed to map IonTorrent data using default parameter settings.[5] Subsequently, the resulting BAM-files were used to obtain total counts of short reads for each gene of the mouse mm10 genome for each sample, respectively, by applying featureCounts which is part of subread-1.4.5.[6] To allocate the gene information needed by featureCounts, the mm10-gtf-file was generated using the UCSC table browser (genome.ucsc.edu/cgi-bin/hgTables) with the options: mouse genome, UCSC known genes, and refFlat table format (July 9^th^ 2015).[7, 8] For each sample analysed total raw reads, mean read length, mapped reads and successfully assigned reads are shown in Table S4 and S5. The tables of total counts of short reads for each sample, the result tables of featureCounts, were imported into R (version 3.1.1).[9] DESeq2 (DESeq2 release 1.6.3[10], Bioconductor version 2.14[11]) was used to analyze the differential gene expression of the CLP- versus sham-treated, with the contrast-option as well as cooksCutoff-filtering set FALSE, independent Filtering set TRUE, log2FoldChange threshold set to 1, and an adjusted p-value cutoff of 0.05. Furthermore, DESeq2 was used to perform a multifactor analysis. This was carried out to identify those genes that are differentially expressed between septic *Nlrp3* KO and septic *Nlrp3* WT mice as well as those genes differentially expressed between septic *Nlrp3* KO mice in comparison to the sham treated *Nlrp3* KO mice considering the interaction term regarding infection and mouse type  http://bioconductor.org/packages/release/bioc/manuals/DESeq2/man/DESeq2.pdf. The genes of interest that were differentially expressed following CLP operation (using the contrast option of DESeq2 for CLP vs. sham) were chosen for further analysis. The upregulated genes as well as the downregulated genes were annotated using the KEGG (Kyoto Encyclopedia of Genes and Genomes) database to identify enriched categories, respectively. For the most relevant enriched categories, heatmaps of their assigned genes were generated. Those heatmaps provide useful information about those genes of these categories that were differently expressed in *Nlrp3* WT and *Nlrp3* KO mice.

**Myoblast culture, differentiation and atrophy assay**.

Cell culture experiments of murine myoblasts (H9c2 cells) were performed as described recently.[2] Differentiation of myoblasts to myotubes was induced at confluence by replacing growth medium (Dulbecco's modification of Eagle medium (DMEM, 1 g/l glucose) (Sigma-Aldrich), 10 % fetal bovine serum (FBS) (PAA Laboratories™), 2 mM L-Glutamine (PAA Laboratories™), supplemented with Penicillin and Streptomycin (both PAA Laboratories™)), with differentiation medium ((DMEM, 1 g/l glucose), 2 % FBS, supplemented with Penicillin and Streptomycin (both PAA Laboratories™)). *Atrophy assay:* myotubes were treated with indicated amounts of recombinant interleukin 1β (ProSpec®) or solvent for 72 hours. An IL-1RA (10 µg/ml, Sigma-Aldrich) and the IκB-kinase inhibitor BMS-345541 (5 µM, Sigma-Aldrich) were added 30 min prior to IL-1β treatment to block the IL-1 receptor and the NF-κB signaling pathway, respectively. The IκB kinase (IKK) inhibitor BMS-345541 (4(2'-aminoethyl)amino-1,8-dimethylimidazo(1,2-a)quinoxaline; CAS-number: 547757-23-3) was used to investigate if IL-1β-mediated effects on gene expression and cell size were NF-κB dependent. IKK phosphorylates IκBα and NF-κB p65, leading to the proteasomal degradation of IκBα and release of active NF-κB[12]. BMS-345541 is a highly selective cell permeable IKK-2 and IKK-1 inhibitor that abolishes IKK-dependent phosphorylation of p65 and of IκBα and subsequent degradation of IκBα and activation of NF-κB.[13] Light microscopic pictures were analyzed using the Leica CTR 6500 microscope and the Leica DFC 360 FX digital camera. From 100 myotubes per condition, three diameters per myotube were measured and averaged using the ImageJ software. The person who performed these measurements was blinded to the specific treatment of the myotubes. Data are presented as frequency-distribution histograms plotting myotubes width against its frequency.

**RNA isolation, cDNA synthesis and quantitative real-time-PCR**.

Total RNA was isolated from cultured H9c2 cells or the interventricular septum of CLP or sham operated mice using TRIzol® Reagent (Invitrogen™, Life Technologies Corporation, CA, USA) and the FastPrep-24™ instrument (MP Biomedicals GmbH) in accordance with manufacturer´s instructions and as recently reported.[14, 15] cDNA synthesis of 1 µg of RNA per sample was carried out by using the SuperScript® First-Strand Synthesis System (Invitrogen™, Life Technologies Corporation, CA, USA) in accordance with manufacturer´s instructions. Quantitative real-time polymerase chain reaction (qPCR) was performed using *Power* SYBR® Green PCR Master Mix (ThermoFisher Scientific) and self-designed primers (for primer sequences see Table S15). PCR reactions were performed in a StepOnePlus™ thermocycler (Applied Biosystems) as described recently using a cDNA standard curve.[1, 14, 16] Expression of specific genes was normalized to the stably expressed reference gene glyceraldehyde-3-phosphate dehydrogenase (*Gapdh*).[1, 14, 16]

**Protein extraction, Western blot assay and EMSA.**

The FastPrep-24™ instrument (MP Biomedicals GmbH) was used to homogenise cardiac tissue in lysis buffer (50 mM Tris, pH 7.5, 150 mM NaCl, 10 % glycerol, 1 % Triton X-100) supplemented with protease inhibitors (cOmplete™ protease inhibitor cocktail; Roche Diagnostics GmbH, Germany) using Micro Packaging Vials with 2.8 mm Precellys ceramic beads (PEQLAB Biotechnology GmbH, Germany). Lysates were cleared by centrifugation at 16.000 g for 20 min at 4 °C. Protein content in the supernatant was quantitated using Pierce® BCA reagent (Thermo Fischer Scientific Inc., MA, USA). Proteins were stored at -80°C. Western blot analysis was performed on protein samples from hearts and cells, as previously described.[2] Briefly, proteins were resolved by SDS-PAGE and transferred onto nitrocellulose membranes (GE Healthcare, Germany). Membranes were blocked with either 5 % skim milk powder or 5 % bovine serum albumin (BSA) dissolved in TBS-T for 1 hour. Following primary antibodies were used: anti-actin (monoclonal, mouse, 1:2000, Sigma-Aldrich, Germany), anti–MyHC (clone MF20, detecting fast and slow myosin, monoclonal, mouse, 1:1000; Sigma-Aldrich, Germany), anti-LC3 (monoclonal, rabbit, 1:1000, NEB, UK), anti-p62 (monoclonal, mouse, 1:1000, Abcam, UK), anti-p65 (mouse, monoclonal, 1:1000, Thermo FisherScientific), anti-phospho p65 (polyclonal, rabbit, 1:500, Abcam UK) antibody. Equal loading was controlled with anti-GAPDH (clone 6C5, monoclonal, mouse, 1:50.000; Millipore GmbH, Germany) antibody. HRP-linked IgG horse anti-mouse, goat anti-rabbit (both 1:2000, Cell Signaling Technology Inc., Danvers, USA) or rabbit anti-goat (1:5000, Abcam, UK) were used as secondary antibody. Proteins were visualized with a chemiluminescence system (SuperSignal® West Pico Chemiluminescent substrate, Thermo Fischer Scientific Inc., MA, USA). NF-κB DNA binding activity in nuclear extracts was detected by an electrophoretic mobility shift assay (EMSA) as described previously.[17, 18]

**References**

1. Lodka D, Pahuja A, Geers-Knorr C, Scheibe RJ, Nowak M, Hamati J, et al. Muscle RING-finger 2 and 3 maintain striated-muscle structure and function. J Cachexia Sarcopenia Muscle. 2016;7:165-80. doi:10.1002/jcsm.12057

2. Fielitz J, Kim M-S, Shelton JM, Latif S, Spencer JA, Glass DJ, et al. Myosin accumulation and striated muscle myopathy result from the loss of muscle RING finger 1 and 3. The Journal of clinical investigation. 2007;117:2486-95. doi:10.1172/jci32827

3. Claycomb WC, Lanson NA, Jr., Stallworth BS, Egeland DB, Delcarpio JB, Bahinski A, et al. HL-1 cells: a cardiac muscle cell line that contracts and retains phenotypic characteristics of the adult cardiomyocyte. Proc Natl Acad Sci U S A. 1998;95:2979-84. doi:10.1073/pnas.95.6.2979

4. Wingett SW, Andrews S. FastQ Screen: A tool for multi-genome mapping and quality control. F1000Res. 2018;7:1338. doi:10.12688/f1000research.15931.2

5. Zeng F, Jiang R, Chen T. PyroHMMsnp: an SNP caller for Ion Torrent and 454 sequencing data. Nucleic Acids Res. 2013;41:e136. doi:10.1093/nar/gkt372

6. Liao Y, Smyth GK, Shi W. featureCounts: an efficient general purpose program for assigning sequence reads to genomic features. Bioinformatics. 2014;30:923-30. doi:10.1093/bioinformatics/btt656

7. Zhao S, Zhang B. A comprehensive evaluation of ensembl, RefSeq, and UCSC annotations in the context of RNA-seq read mapping and gene quantification. BMC genomics. 2015;16:97. doi:10.1186/s12864-015-1308-8

8. Wu PY, Phan JH, Wang MD. Assessing the impact of human genome annotation choice on RNA-seq expression estimates. BMC Bioinformatics. 2013;14 Suppl 11:S8. doi:10.1186/1471-2105-14-S11-S8

9. Dillies MA, Rau A, Aubert J, Hennequet-Antier C, Jeanmougin M, Servant N, et al. A comprehensive evaluation of normalization methods for Illumina high-throughput RNA sequencing data analysis. Brief Bioinform. 2013;14:671-83. doi:10.1093/bib/bbs046

10. Love MI, Huber W, Anders S. Moderated estimation of fold change and dispersion for RNA-seq data with DESeq2. Genome Biol. 2014;15:550. doi:10.1186/s13059-014-0550-8

11. Anders S, McCarthy DJ, Chen Y, Okoniewski M, Smyth GK, Huber W, et al. Count-based differential expression analysis of RNA sequencing data using R and Bioconductor. Nat Protoc. 2013;8:1765-86. doi:10.1038/nprot.2013.099

12. Deng L, Wang C, Spencer E, Yang L, Braun A, You J, et al. Activation of the IkappaB kinase complex by TRAF6 requires a dimeric ubiquitin-conjugating enzyme complex and a unique polyubiquitin chain. Cell. 2000;103:351-61.

13. Burke JR, Pattoli MA, Gregor KR, Brassil PJ, MacMaster JF, McIntyre KW, et al. BMS-345541 is a highly selective inhibitor of I kappa B kinase that binds at an allosteric site of the enzyme and blocks NF-kappa B-dependent transcription in mice. J Biol Chem. 2003;278:1450-6. doi:10.1074/jbc.M209677200

14. Du Bois P, Pablo Tortola C, Lodka D, Kny M, Schmidt F, Song K, et al. Angiotensin II Induces Skeletal Muscle Atrophy by Activating TFEB-Mediated MuRF1 Expression. Circ Res. 2015;117:424-36. doi:10.1161/CIRCRESAHA.114.305393

15. Zhu X, Kny M, Schmidt F, Hahn A, Wollersheim T, Kleber C, et al. Secreted Frizzled-Related Protein 2 and Inflammation-Induced Skeletal Muscle Atrophy. Crit Care Med. 2017;45:e169-e83. doi:10.1097/CCM.0000000000002056

16. Wollersheim T, Woehlecke J, Krebs M, Hamati J, Lodka D, Luther-Schroeder A, et al. Dynamics of myosin degradation in intensive care unit-acquired weakness during severe critical illness. Intensive Care Med. 2014;40:528-38. doi:10.1007/s00134-014-3224-9

17. Krappmann D, Emmerich F, Kordes U, Scharschmidt E, Dorken B, Scheidereit C. Molecular mechanisms of constitutive NF-kappaB/Rel activation in Hodgkin/Reed-Sternberg cells. Oncogene. 1999;18:943-53. doi:10.1038/sj.onc.1202351

18. Hahn A, Kny M, Pablo-Tortola C, Todiras M, Willenbrock M, Schmidt S, et al. Serum amyloid A1 mediates myotube atrophy via Toll-like receptors. J Cachexia Sarcopenia Muscle. 2020;11:103-19. doi:10.1002/jcsm.12491

**Supporting Tables**

**Table S1.** Primer pairs for genotyping of *Nlrp3* KO and *Nlrp3* WT mice.

| **Name** | **Sequence (5’ – 3’)** |
| --- | --- |
| **Mm_*Nlrp3*_WT forward** | TCA AGC TAA GAG AAC TTT CTG |
| **Mm_*Nlrp3*_WT reverse** | ACA CTC GTC ATC TTC AGC A |
| **Mm_*Nlrp3*_KO forward** | TCA AGC TAA GAG AAC TTT CTG |
| **Mm_*Nlrp3*_KO reverse** | AAG TCG TGC TGC TTC ATG T |

Nlrp3 indicates NOD-like receptor family, pyrin domain containing protein-3; WT, wild type; KO, knockout; Mm, Mus musculus.

**Table S2. Body and organ weights 96 hours after CLP or sham surgery**

|  | *Nlrp3* WT  sham  (n=18) | *Nlrp3* WT  CLP  (n=12) | *Nlrp3* KO  sham  (n=8) | *Nlrp3* KO  CLP  (n=16) | *p-*value  KO CLP vs. WT CLP |
| --- | --- | --- | --- | --- | --- |
| BW [g] | 21.9 ± 4.12 | 18.93 ± 2.32* | 22.41 ± 1.43 | 20.73 ± 1.99* | n.s. |
| TL [mm] | 16.06 ± 0.84 | 16.41 ± 0.44 | 16.34 ± 0.54 | 16.29 ± 0.34 | n.s. |
| BW / TL [g/mm] | 1.36 ± 0.21 | 1.15 ± 0.12*** | 1.37 ± 0.08 | 1.27 ± 0.12* | * |
| HW [mg] | 102.7 ± 24.3 | 89.7 ± 10.3* | 109.6 ± 9.7 | 105.8 ± 17.3 | ** |
| HW / TL [mg/mm] | 6.34 ± 1.27 | 5.46 ± 0.57* | 6.71 ± 0.6 | 6.49 ± 1.05 | ** |
| LuW [mg] | 130.5 ± 21.4 | 122.2 ± 9.0 | 127.9 ± 7.8 | 122.5 ± 11.6 | n.s. |
| LuW / TL [mg/mm] | 8.10 ± 1.08 | 7.45 ± 0.52* | 7.83 ± 0.56 | 7.52 ± 0.72 | n.s. |
| LiW [mg] | 1254.9 ± 241.5 | 910.2 ± 160.0*** | 1255.3 ± 151.6 | 1197.7 ± 166.7 | *** |
| LiW / TL [mg/mm] | 77.70 ± 12.29 | 55.36 ± 9.16*** | 76.85 ± 9.85 | 73.49 ± 10.31 | *** |

BW indicates body weight 96 hours after surgery; HW, heart weight; LuW, lung weight; LiW, liver weight; TL, tibia length; CLP, cecal ligation and puncture. **p*≤0.05; ****p*≤0.001; n.s. = not significant.

**Table S3. Data of transthoracic echocardiography**

|  | *Nlrp3* WT  sham  (n=13) | *Nlrp3* WT  CLP  (n=13) | *Nlrp3* KO  sham  (n=18) | *Nlrp3* KO  CLP  (n=18) | *p* CLP  (WT vs. KO) |
| --- | --- | --- | --- | --- | --- |
| SV [µl] | 33.09 ± 7.22 | 24.57 ± 8.68* | 28.30 ± 8.12 | 29.94 ± 8.96 | n.s. |
| CO [ml/min] | 14.61 ± 4.27 | 11.14 ± 3.33* | 13.68 ± 3.53 | 16.12 ± 4.61 | * |
| IVSthd [mm] | 0.77 ± 0.10 | 0.82 ± 0.1 | 0.81 ± 0.09 | 0.85 ± 0.13 | n.s. |
| IVSths [mm] | 1.19 ± 0.22 | 1.24 ± 0.15 | 1.21 ± 0.21 | 1.35 ± 0.24 | n.s. |
| LVPWthd [mm] | 0.77 ± 0.10 | 0.82 ± 0.10 | 0.83 ± 0.09 | 0.86 ± 0.14 | n.s. |
| LVPWths [mm] | 1.16 ± 0.23 | 1.26 ± 0.13 | 1.27 ± 0.23 | 1.41 ± 0.24 | * |
| LVIDd [mm] | 4.17 ± 0.43 | 3.29 ± 0.65*** | 3.83 ± 0.45 | 3.67 ± 1.51 | n.s. |
| LVIDs [mm] | 2.74 ± 0.61 | 2.06 ± 0.69* | 2.52 ± 0.55 | 1.95 ± 0.62* | n.s. |
| FS [%] | 34.92 ± 9.66 | 38.80 ± 9.09 | 34.98 ± 7.65 | 46.97 ± 9.76*** | ** |
| LVEF [%] | 62.03 ± 10.69 | 65.82 ± 11.42 | 63.04 ± 8.82 | 71.14 ± 7.98** | ** |
| E [mm/s] | 750 ± 132 | 522 ± 200*** | 709 ± 152 | 639 ± 165 | * |
| A [mm/s] | 406.6 ± 78.6 | 321.4 ± 96.6* | 455.1 ± 109.0 | 398.1 ± 124.9 | n.s. |
| E/A | 1.89 ± 0.37 | 1.54 ± 0.27* | 1.59 ± 0.26 | 1.66 ± 0.30 | ** |

SV indicates stroke volume; CO, cardiac output; IVSthd indicates interventricular septal thickness at diastole; IVSths, interventricular septal thickness at systole; LVPWthd, left ventricular posterior wall thickness at diastole; LVPWths, left ventricular posterior wall thickness at systole; LVIDd, left ventricular end-diastolic dimension; LVIDs, left ventricular end-systolic dimension; FS, fractional shortening; LVEF, left ventricular ejection fraction; E/A, ratio of peak flow velocity across the mitral annulus during early and late diastole; CLP, cecal ligation and puncture. **p*≤0.05; ***p*≤0.01; ****p*≤0.001; n.s. = not significant.

**Table S4.** Total raw reads, mean read length, mapped reads for each sample analysed.

|  | Total raw reads | Mean read length | Mapped reads | Mapped reads (%) |
| --- | --- | --- | --- | --- |
| WT sham #1 | 28,051,649 | 114 | 27,082,227 | 96.5 |
| WT sham #2 | 19,583,428 | 121 | 19,074,376 | 97.4 |
| WT sham #3 | 25,495,900 | 121 | 24,769,254 | 97.1 |
| WT CLP #1 | 16,001,716 | 116 | 15,561,613 | 97.2 |
| WT CLP #2 | 21,262,597 | 117 | 20,631,822 | 97.0 |
| WT CLP #3 | 19,175,465 | 126 | 18,430,847 | 96.1 |
| KO sham #1 | 20,054,317 | 118 | 19,335,726 | 96.4 |
| KO sham #2 | 22,854,147 | 120 | 21,890,061 | 95.8 |
| KO sham #3 | 25,645,938 | 129 | 24,951,206 | 97.3 |
| KO CLP #1 | 25,361,630 | 133 | 24,712,342 | 97.4 |
| KO CLP #2 | 19,754,946 | 135 | 19,098,725 | 96.7 |
| KO CLP #3 | 12,632,948 | 125 | 12,273,502 | 97.2 |

**Table S5.** Successfully assigned reads for each sample analysed.

|  | Total mapped reads | Successfully  assigned reads | Successfully  assigned reads (%) |
| --- | --- | --- | --- |
| *Nlrp3* WT sham #1 | 27,082,227 | 17,210,550 | 63.5 |
| *Nlrp3* WT sham #2 | 19,074,376 | 11,297,239 | 59.2 |
| *Nlrp3* WT sham #3 | 24,769,254 | 14,220,036 | 57.4 |
| *Nlrp3* WT CLP #1 | 15,561,613 | 9,710,032 | 62.4 |
| *Nlrp3* WT CLP #2 | 20,631,822 | 12,649,924 | 61.3 |
| *Nlrp3* WT CLP #3 | 18,430,847 | 10,637,906 | 57.7 |
| *Nlrp3* KO sham #1 | 19,335,726 | 10,507,022 | 54.3 |
| *Nlrp3* KO sham #2 | 21,890,061 | 12,585,663 | 57.5 |
| *Nlrp3* KO sham #3 | 24,951,206 | 14,584,620 | 58.5 |
| *Nlrp3* KO CLP #1 | 24,712,342 | 15,181,409 | 61.4 |
| *Nlrp3* KO CLP #2 | 19,098,725 | 11,168,174 | 58.5 |
| *Nlrp3* KO CLP #3 | 12,273,502 | 7,606,200 | 62.0 |

**Table S6.** ≥ 2-fold upregulated genes in hearts of CLP vs. sham treated *Nlrp3* WT mice (.xls table; Table S5_wt_CLP vs wt_sham_FC2.xlsx)

**Table S7.** ≥ 2-fold upregulated genes in hearts of CLP vs. sham treated *Nlrp3* KO mice (.xls table; Table S6_ko_CLP vs ko_sham_FC2.xlsx)

**Table S8.** Twentyone genes upregulated in both septic *Nlrp3* WT and septic *Nlrp3* KO hearts compared to the respective sham group

| ID | Name |
| --- | --- |
| Lilrb4 | Leukocyte immunoglobulin-like receptor subfamily B member 4 |
| Rpl30-ps3 | Ribosomal protein L30, pseudogene 3 |
| Gm12468 | Predicted |
| Gm11703 | Predicted |
| Hcls1 | Hematopoietic Cell-Specific Lyn Substrate 1, |
| Rpl30 | Ribosomal Protein L30 |
| Rpl13-ps1 | Ribosomal protein L13, pseudogene 1 |
| Rps19-ps7 | Ribosomal protein S19, pseudogene 7 |
| Rpl13a-ps1 | Ribosomal protein 13A, pseudogene 1 |
| Rps18-ps1 | Ribosomal protein S18, pseudogene 1 |
| Gm10443 | Predicted |
| Rps19 | 40S ribosomal protein S19 |
| Hist1h4h | Histone cluster 1 H4 family member h |
| Gm8731 | Predicted |
| Gm3699 | Predicted |
| Gm6023 | Predicted |
| Gm8692 | Predicted |
| Gm5121 | Predicted |
| Gm10736 | Predicted |
| Gm13268 | Predicted |
| Gm16238 | Predicted |

**Table S9.** ≥ 2-fold downregulated genes in hearts of CLP vs. sham treated *Nlrp3* WT mice (.xls table; Table S8_wt_CLP vs wt_sham_FC-2.xlsx)

**Table S10.** ≥ 2-fold downregulated genes in hearts of CLP vs. sham treated *Nlrp3* KO mice (.xls table; Table S9_ko_CLP vs ko_sham_FC-2.xlsx)

**Table S11.** Five genes downregulated in both septic *Nlrp3* WT and septic *Nlrp3* KO hearts compared to the respective sham group

| ID | Name |
| --- | --- |
| Nrep | Neuronal regeneration related protein |
| Pxdn | Peroxidasin |
| Efnb3 | Ephrin B3 |
| Fam131a | Family With Sequence Similarity 131 Member A |
| Sox7 | SRY-box 7 |

**Table S12. KEGG pathway analysis of significantly upregulated genes in septic *Nlrp3* WT compared to sham treated *Nlrp3* WT hearts.**

**Table S13. KEGG pathway analysis of significantly downregulated genes in septic *Nlrp3* WT compared to sham treated *Nlrp3* WT hearts.**

**Table S14. KEGG pathway analysis of significantly upregulated genes in septic *Nlrp3* KO compared to sham treated *Nlrp3* KO hearts.**

**Table S15.** Primer pairs for quantitative real-time-PCR are shown.

| **Name** | **Sequence (5’- 3’)** |
| --- | --- |
| **Mm_*Fbxo32* forward** | AGT GAG GAC CGG CTA CTG TG |
| **Mm_*Fbxo32* reverse** | GAT CAA ACG CTT GCG AAT CT |
| **Mm_*Gapdh* forward** | ATG GTG AAG GTC GGT GTG A |
| **Mm_*Gapdh* reverse** | AAT CTC CAC TTT GCC ACT GC |
| **Mm_*Il1b* forward** | AGT TGA CGG ACC CCA AAA G |
| **Mm_*Il1b* reverse** | AGC TGG ATG CTC TCA TCA GG |
| **Mm_*Il6* forward** | GCT ACC AAA CTG GAT ATA ATC AGG A |
| **Mm_*Il6* reverse** | CCA GGT AGC TAT GGT ACT CCA GAA |
| **Mm_*Myh2* forward** | AAC TCC AGG CAA AAG TGA AAT C |
| **Mm_*Myh2* reverse** | TGG ATA GAT TTG TGT TGG ATT GTT |
| **Mm_*Myh4* forward** | GGG AAC ATG AAA TTC AAG CAA |
| **Mm_*Myh4* reverse** | ATA GGC AGC CTT GTC AGC AA |
| **Mm_*Myh7* forward** | CGC ATC AAG GAG CTC ACC |
| **Mm_*Myh7* reverse** | CTG CAG CCG CAG TAG GTT |
| **Mm_*Nlrp3* forward** | CCC TTG GAG ACA CAG GAC TC |
| **Mm_*Nlrp3* reverse** | GAG GCT GCA GTT GTC TAA TTC C |
| **Mm_*Trim63* forward** | CCT GCA GAG TGA CCA AGG A |
| **Mm_*Trim63* reverse** | GGC GTA GAG GGT GTC AAA CT |
| **Rn_*Fbxo32* forward** | GAA GAC CGG CTA CTG TGG AA |
| **Rn_*Fbxo32* reverse** | ATC AAT CGC TTG CGG ATC T |
| **Rn_*Gapdh* forward** | CAA GGT CAT CCA TGA CAA CTT TG |
| **Rn_*Gapdh* reverse** | GGG CCA TCC ACA GTC TTC TG |
| **Rn_*Il6* forward** | CCC TTC AGG AAC AGC TAT GAA |
| **Rn_*Il6* reverse** | ACA ACA TCA GTC CCA AGA AGG |
| **Rn_*Nlrp3* forward** | GCT GTG TGA GGC ACT CCA G |
| **Rn_*Nlrp3* reverse** | GAA ACA GCA TTG ATG GGT CA |
| **Rn_*Trim63* forward** | AGG ACT CCT GCC GAG TGA C |
| **Rn_*Trim63* reverse** | TTG TGG CTC AGT TCC TCC TT |

Trim63 indicates Tripartite motif 63; Fbxo32, F-Box only 32; Gapdh, glyceraldehyde-3-phosphate dehydrogenase; Il-6, interleukin 6; Il-1b, interleukin 1β; Myh, myosin heavy chain; Nlrp3, NOD-like receptor family, pyrin domain containing 3; Mm, Mus musculus ; Rn, rattus norwegicus.

**Supporting Figures**

**Figure S1. *Nlrp3* KO mice are protected from septic cardiomyopathy.** 12-16-week-old male *Nlrp3* KO and WT mice were subjected to CLP or sham surgery. Haematoxylin and eosin (**A, B**) and trichrome (**C, D**) staining of histological sections from hearts of sham and CLP operated WT and *Nlrp3* KO mice at 96 hours after surgery, as indicated, are shown. Gross morphology (**A, C**; Scale bar = 1 mm) and higher magnifications (**B, D**; Scale bar = 100 µm) are shown.

**Figure S2. Genes involved in “Immune System Process” are less activated in hearts of septic *Nlrp3* KO mice.** Heatmap of normalized expression values of genes involved in Immune System Process. Genotypes and treatments as well as the z-score are indicated.

**Figure S3. IL-1β causes atrophy of cardiomyocytes in a dose- and time-dependent manner.** Differentiated H9c2 myotubes were treated with increasing amounts of recombinant IL-1β (10 ng/ml, 20 ng/ml, 50 ng/ml) or vehicle for 72 hours. **A**, Representative light microscopy pictures. Scale bar = 250 µm. **B**, Frequency distribution histograms of cell width of vehicle and IL-1β (10 ng/ml, 20 ng/ml and 50 ng/ml) treated myotubes, n = 100 cells per condition. **C**, Mean myotube width. **D**, Differentiated H9c2 myotubes were treated with recombinant IL-1β (50 ng/ml) or vehicle for 3 hours, 6 hours, 12 hours, 24 hours, 48 hours and 72 hours, respectively. Western blot analysis of isolated proteins using anti-MyHC slow is shown. GAPDH was used as loading control. Data are presented as mean ± SEM. ***p*≤0.01, ****p*≤0.001, *****p*≤0.0001.
